# Identification of small RNAs abundant in *Burkholderia cenocepacia* biofilms reveal putative regulators with a potential role in carbon and iron metabolism

**DOI:** 10.1038/s41598-017-15818-3

**Published:** 2017-11-15

**Authors:** Andrea Sass, Sanne Kiekens, Tom Coenye

**Affiliations:** Department of Pharmaceutical Microbiology, Ghent University, Ghent, Belgium

## Abstract

Small RNAs play a regulatory role in many central metabolic processes of bacteria, as well as in developmental processes such as biofilm formation. Small RNAs of *Burkholderia cenocepacia*, an opportunistic pathogenic beta-proteobacterium, are to date not well characterised. To address that, we performed genome-wide transcriptome structure analysis of biofilm grown *B. cenocepacia* J2315. 41 unannotated short transcripts were identified in intergenic regions of the *B. cenocepacia* genome. 15 of these short transcripts, highly abundant in biofilms, widely conserved in *Burkholderia* sp. and without known function, were selected for in-depth analysis. Expression profiling showed that most of these sRNAs are more abundant in biofilms than in planktonic cultures. Many are also highly abundant in cells grown in minimal media, suggesting they are involved in adaptation to nutrient limitation and growth arrest. Their computationally predicted targets include a high proportion of genes involved in carbon metabolism. Expression and target genes of one sRNA suggest a potential role in regulating iron homoeostasis. The strategy used for this study to detect sRNAs expressed in *B. cenocepacia* biofilms has successfully identified sRNAs with a regulatory function.

## Introduction

*Burkholderia cenocepacia* is a member of the *Burkholderia cepacia* complex (Bcc), a group of closely related beta-proteobacteria^1^ widely occurring in the environment, particularly in the rhizosphere^2^. Many Bcc species are also opportunistic pathogens, able to infect cystic fibrosis patients and immunocompromised individuals^2,3^. Infections with Bcc bacteria are very difficult to treat due to their high innate antimicrobial resistance. Their ability to form biofilms adds to their recalcitrance to antimicrobial treatment^4^.

Biofilm formation is a highly regulated developmental process, and the post-transcriptional level of regulation plays a large role in this process. In particular, small non-coding regulatory RNAs (sRNAs) have been identified as important factors in the regulatory network of biofilm formation^5,6^.

sRNAs are typically 50–500 nucleotides in size and can regulate gene expression by interacting with other RNAs or with proteins^7,8^. Most known sRNAs interact with mRNAs, by incomplete base-pairing to short target sequences, and for this the Hfq protein is required as chaperone^9,10^. These sRNAs regulate protein expression by altering the rate of translation or of mRNA degradation, and they are usually trans-acting: they interact with multiple targets at distinct genome locations^11^. *B. cenocepacia* possesses two Hfq homologues^12^, suggesting that sRNAs and their mRNA-binding mechanism of regulating protein expression play a role in this bacterium.

*B. cenocepacia* J2315, like all Bcc bacteria, has a relatively large genome of >8 Mb with a high GC-content (66.9%), with >7200 coding sequences and a large number of mobile genetic elements and genomic islands^12^. The genome has a multi-replicon structure: the largest replicon (3.87 Mb) harbours most of the major housekeeping genes, whereas the two smaller replicons (3.22 Mb and 0.88 Mb) and the plasmid (0.09) harbour fewer core and more accessory functions such as antibiotic and antifungal production^12^. The *B. cenocepacia* J2315 reference genome annotation includes six rRNA operons, 74 tRNAs and 21 other small non-coding RNAs. Among these are tmRNA, RNase P and the signal recognition particle; as well as 14 riboswitches and four catalytic introns^12^. A number of un-annotated sRNAs have been identified in *B. cenocepacia* J2315 by RNA-Sequencing (RNA-Seq) and Northern blotting^13^, by microarray analysis^14–16^, binding to Hfq-protein^17^, and by computational prediction^18^.

**Figure 1:**
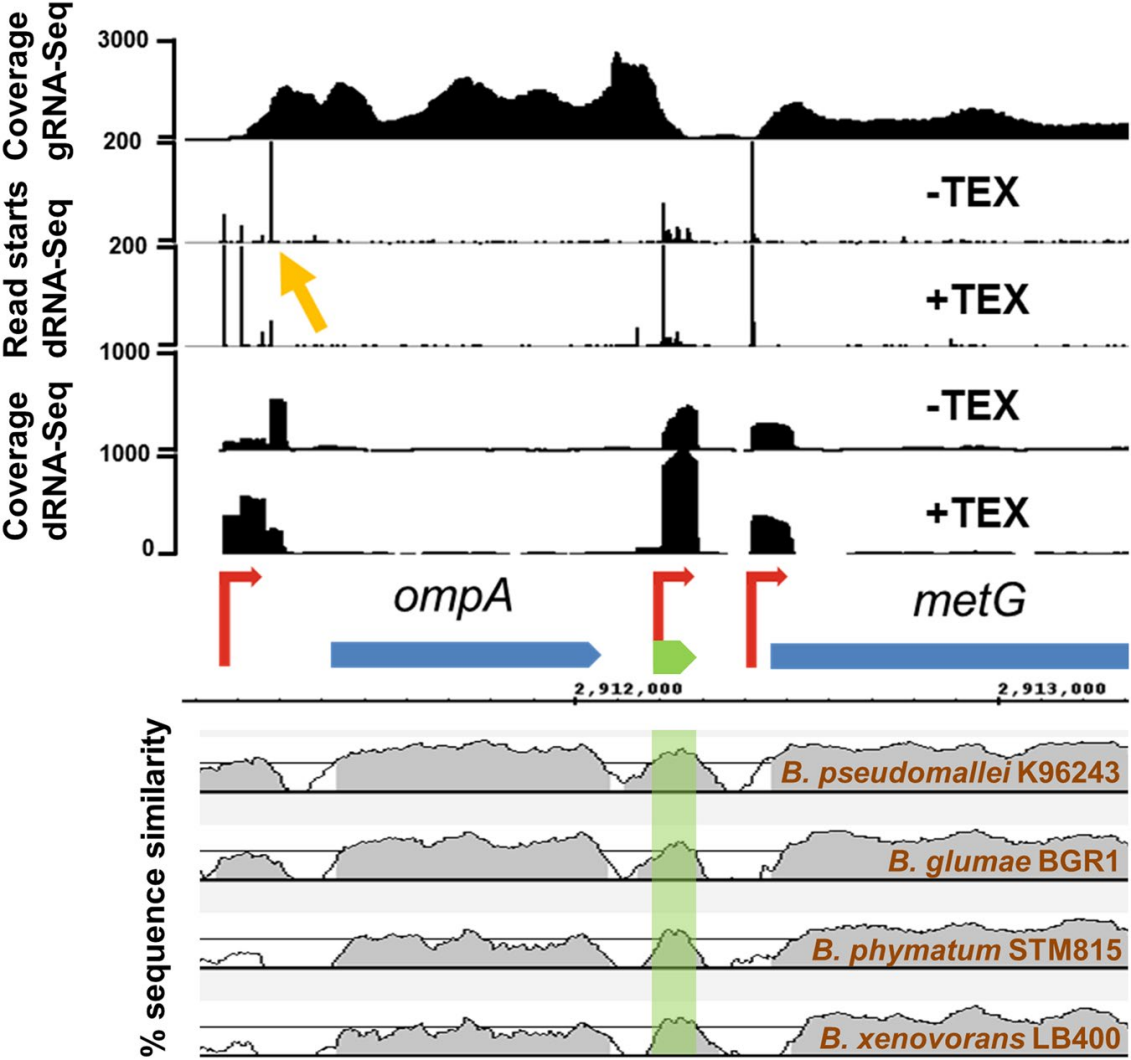
Genome location and conservation of candidate sRNA ncS16. Upper panel: coverage and number of gene starts from gRNA-Seq and dRNA-Seq. Blue: annotated CDS; red: TSS; green: sRNA. TSS are characterised by higher coverage in TEX-treated sample. The candidate sRNA ncS16 has a dedicated TSS, located in the 3′UTR of BCAL2645 (*ompA*). The 5′UTR of BCAL2645 is processed (yellow arrow). Lower panel: Sequence conservation. ncS16 is more conserved than directly adjacent sequences. Alignments are arranged, from top to bottom, by increasing phylogenetic distance. Lines represent percent similarity, in grey: similarity ≥70%. Location of ncS16 is shaded in green.

In a previous study, we investigated the transcriptome structure of *B. cenocepacia* J2315 biofilms by transcription start site (TSS) mapping using differential RNA-Sequencing (dRNA-Seq)^19^. For dRNA-Seq, a RNA subsample is treated with a 5′-phosphate dependent exonuclease (Terminator exonuclease, TEX). Native RNAs carry a 5′ triphosphate and are protected from degradation by TEX, RNAs which have been cleaved ( = processed) carry a 5′ monophosphate and are depleted by TEX-treatment. TSS can then be identified in dRNA-Seq data as genome loci with abrupt increase in read coverage, enriched in the +TEX library^20^ (Fig. 1) compared to untreated RNA. dRNA-Seq could confirm expression of most annotated small RNAs^19^, showing that short transcripts of *B. cenocepacia* J2315 are detected with this technique. The dRNA-Seq approach can also detect RNA processing sites. For example, the start of 6 S RNA of *B. cenocepacia* J2315 was depleted in the +TEX library^19^, showing that it is processed, as demonstrated for *E. coli* 6S RNA^21^.

In the present study, we present a comprehensive analysis of short transcripts located in intergenic regions of the *B. cenocepacia* J2315 genome, expressed in biofilms, as independently transcribed sRNAs or as part of untranslated regions of mRNA (UTRs).

## Results

### Identification of independently transcribed sRNAs and of short 5′UTRs

Most known sRNAs are encoded in intergenic regions (IGR), and can also be found in 3′UTRs and 5′UTRs^22,23^. Therefore all intergenic TSS in *B. cenocepacia* J2315 were inspected for short transcripts, regardless of position relative to annotated coding sequences (CDS).

To distinguish independently transcribed sRNAs from 5′UTRs of genes and from longer antisense transcripts, the dRNA-Seq dataset was compared to conventional RNA-Seq data (“global” RNA-Seq, gRNA-Seq)^19^, derived from the same growth condition. Sequences appearing to be short or missing in gRNA-Seq data and without continuous coverage into a 3′-adjacent CDS with the same orientation were reported as sRNAs (Figs 1, S1).

**Figure 2:**
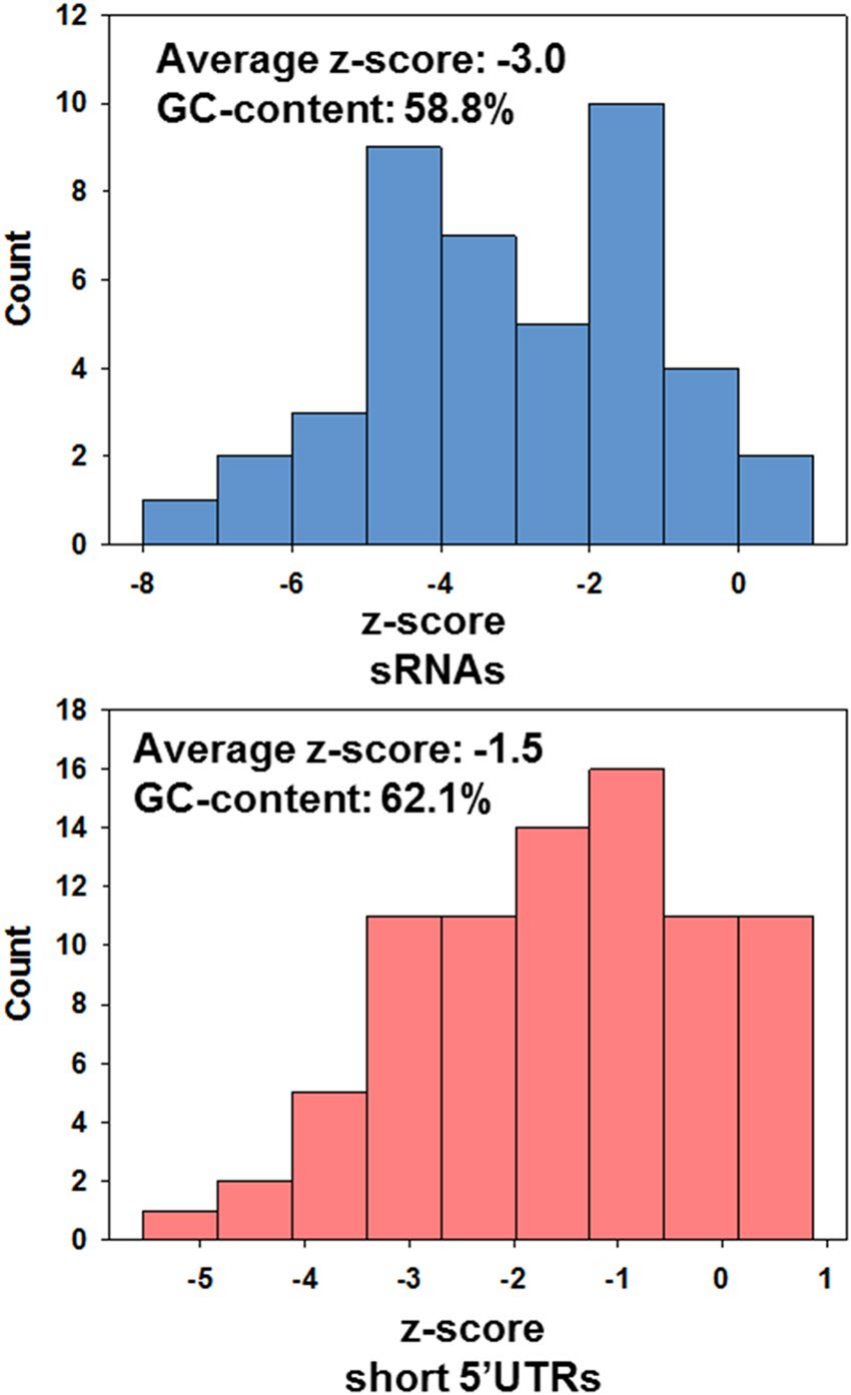
Distribution of z-scores for MFE for sRNAs and short 5′UTRs. The z-score is used as a measure for structural significance. It allows comparing the actual MFE of a given sequence to the distribution of MFEs of randomly mononucleotide shuffled sequences with the same nucleotide composition. More negative values denote a higher structural significance.

In this manner, 148 TSS for sRNAs were discovered in IGRs of the *B. cenocepacia* genome (Table S1). 41 of these transcripts were associated with a rho-independent terminator, their lengths ranged from 57 to 514 nt and their average GC-content was 58.8% (excluding the AT-rich stretch of nucleotides following the terminator, Table S2). 86% of these sRNAs have a z-score for minimum folding energy (MFE) <−1 (Fig. 2).

15 RNAs, transcribed in the same orientation as their downstream adjacent genes, and previously classified as sRNAs (based on their differential expression compared to the downstream CDS) in *B. cenocepacia* ^13^, *B. pseudomallei* ^24^ or *B. thailandensis* ^25^ were classified as 5′UTRs in the present study (Table S3).

**Figure 3:**
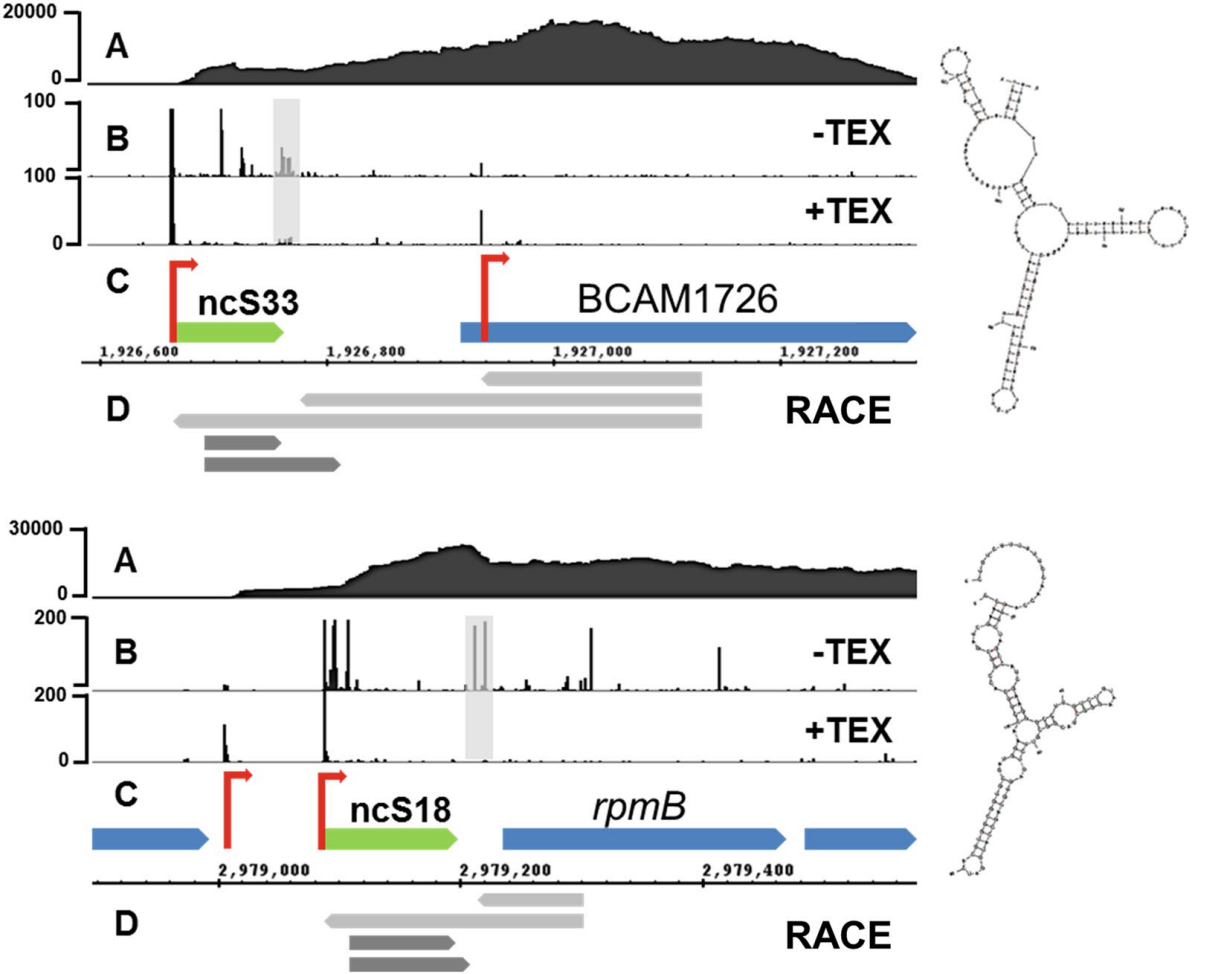
RACE analysis of two processed 5′UTRs, ncS33 (top) and ncS18 (bottom). (**A**) gRNA-Seq reveals continuous coverage from TSS across CDS. (**B**) Read starts in dRNA-Seq data reveal TSS and processing sites (grey box). (**C**) Sequence elements. Green: sRNA, blue: annotated CDS, yellow: predicted rho-independent terminators, red: TSS (**D**) RACE sequence results. All 3′RACE sequences end near predicted processing site (screening of >40 clones: no longer sequences found), whereas approx. 50% of 5′RACE sequences extend across the processing site to the TSS. Left: Secondary structure of ncS33 and ncS18.

We investigated the 5′UTRs ncS18 and ncS33, previously described as sRNAs^13,19^, with 3′ and 5′RACE, to evaluate the proportion of short transcripts compared to full length transcripts derived from their TSS (Fig. 3). Both are highly expressed and appear to be processed. 3′RACE from the 5′UTR towards the CDS resulted exclusively in short sequences which did not extend into the CDS. For ncS33, RACE results indicated two processed species with defined length, for ncS18 the 3′end was less defined (Doc. S1). We used the species associated with the most 3′RACE sequences for structural analysis. ncS18 and ncS33 are both highly structured (Fig. 3), and ncS33 has a negative z-score of −2.5 (Table S3). 5′RACE from within the CDS towards the TSS resulted in short sequences ending near the processing site and in longer sequences extending to the TSS in approximately equal numbers. Both ncS18 and ncS33 are therefore 5′UTRs with a dual nature: 5′end of long mRNA and sRNA. ncS18 was co-regulated with its downstream gene (BCAL2714) across four growth conditions (Fig. S2), while ncS33 was differentially regulated from its downstream gene (BCAM1726, Fig. S2).

Inspection of all 5′UTRs >72 nt (the previously determined average length of 5′UTRs)^19^ identified 82 5′UTRs which produced short transcripts (Table S3), either due to processing, or to attenuation. Attenuation in this context was defined as marked decrease in coverage in gRNA-Seq data, associated with a terminator structure. For the sake of distinguishing them from sRNAs, they are referred to in this study as “short 5′UTRs”. Their length ranged from to 54 to 607 nt, 58% have a z-score for MFE <−1 (Fig. 2). The GC-content of all short 5′UTRs combined was 62.1%. No correlation was found between GC-content and z-score, for neither group of short transcripts.

### Novel candidate regulatory sRNAs highly expressed in biofilms

**Figure 4:**
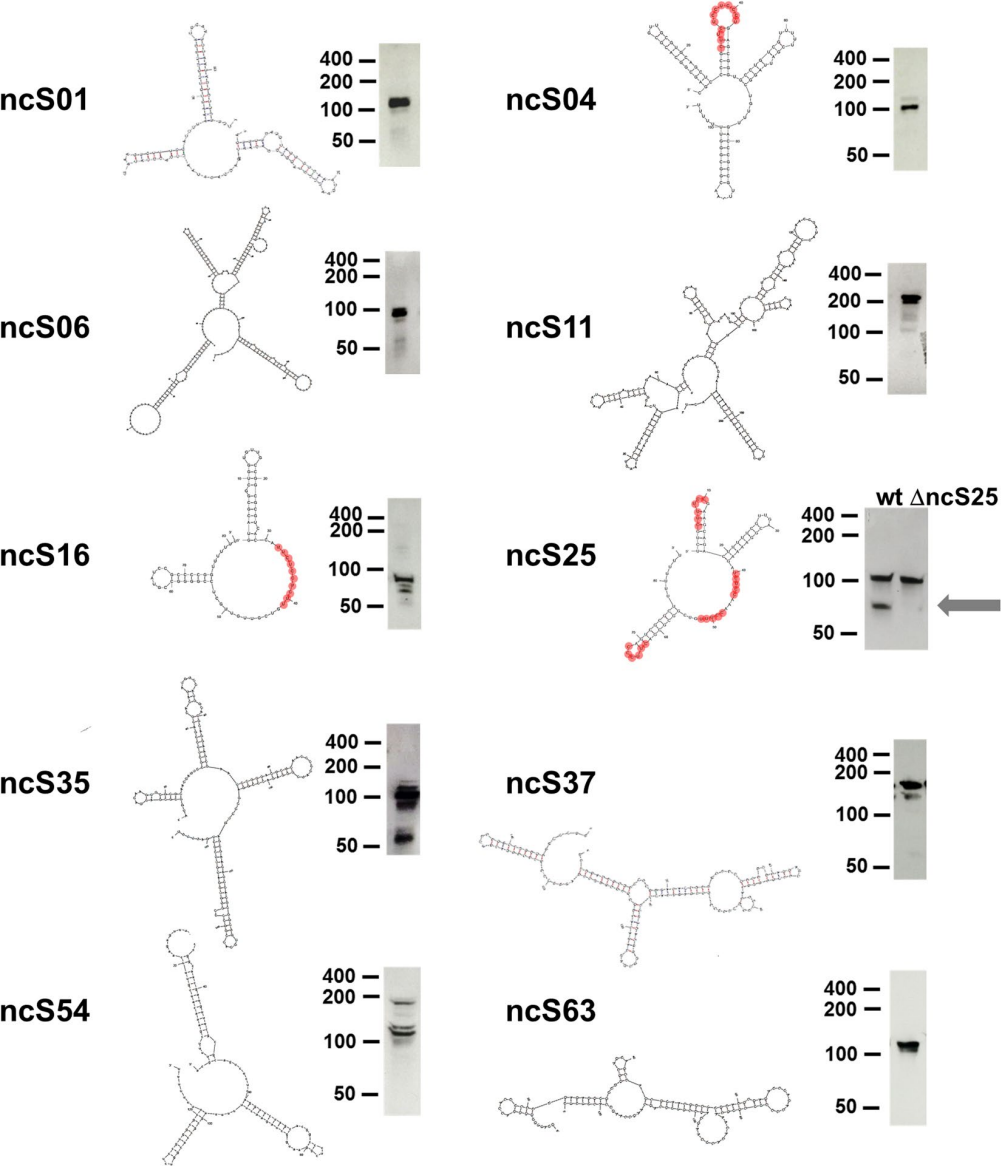
Predicted secondary structure of candidate regulatory sRNAs confirmed by Northern blotting. Secondary structures were predicted with mfold. Northern blot images are from biofilm samples, y-axis depicts sRNA size, in nucleotides, estimated by RNA ladders run in parallel. CU-rich sequence stretches are indicated in red. For ncS25, a deletion mutant was included in Northern analysis, the band not present in the mutant (grey arrow) corresponds in size to predictions. Full size Northern blot images are presented in Fig. S6.

To identify regulatory sRNAs which could play a role in biofilm formation, independently transcribed sRNAs with a predicted rho-independent terminator and without an open reading frame were further filtered for strong expression (arbitrary cut-off: ≥250 read starts at TSS), low z-score (<−1) and conservation in 13 sequenced Bcc strains. 14 sRNAs fulfilled these criteria and were chosen for more in-depth analysis, these were designated “candidate sRNAs” (Table 1, Fig. 4, Fig. S1). One short transcript, located in a 3′UTR and co-transcribed with its preceding CDS, was also included in this shortlist: sRNAs can be produced from 3′-ends of mRNAs^26^ and the latter short transcript was characterised by a processing site followed by a strong increase in coverage (Fig. S1). Comparison of candidate sRNAs with the Rfam database resulted in few hits (Table 1) for sRNAs with confirmed expression, none of which conveyed any information about a putative function.

**Table 1:** Candidate sRNAs strongly expressed in *B. cenocepacia* J2315 biofilms.

Name	Replicon	Genome position	Strand	Length (nt)	MFE^3)^	z-score	Coverage at TSS	Rfam ID	Alternative names
ncS01	1	20581..20691	+	111^1)2)^	−45.3	−4.3	829		
ncS02	1	42693..42890	−	198^1)2)^	−81.1	−1.1	7533	RF02423	Bp1_781^24^
ncS03	1	221314..221372	+	59^2)^	−27.5	−7.2	1141	RF02278	Toxic small RNA^62^
ncS04	1	292949..293053	+	105^1)2)^	−52.7	−5.5	336		anti-*hemB* ^24^
ncS05	1	479440..479506	−	67^2)^	−30.3	−6.4	2588	RF02278	Toxic small RNA^62^
ncS06	1	603652..603910	+	259^1)^	−102.0	−2.4	213		BTH_s1^25^, ncRNA7^13^
ncS54	1	1179680..1179807	+	128^2)^	−48.0	−4.3	269		
ncS11	1	2545296..2545503	−	208^1)^	−82.4	−2.3	2010		ncRNA13^13^
ncS63	1	2548559..2548685	+	127^2)^	−41.6	−1.0	1295*		BTH_s39^25^
ncS16	1	2912201..2912283	+	83^1)2)^	−28.6	−1.7	335		
ncS25	1	3297991..3298074	−	85^2)^	−29.6	−4.7	968		
ncS27	1	3666557..3666648	−	92^1)2)^	−37.8	−5.1	1957	RF02278	Toxic small RNA^62^
ncS35	2	2304213..2304378	−	166^1)2)^	−77.8	−4.6	8236		
ncS37	2	2568766..2568918	+	153^2)^	−79.0	−3.2	882		
ncS62	2	2089713..2089769	+	57^2)^	−22.6	−5.5	312	RF02278	Toxic small RNA^62^

1) 3′end confirmed by RACE; 2) 3′end defined in dRNA-Seq data; 3) MFE, minimum folding energy: initial ΔG derived from mfold; *coverage at processing site.

Twelve candidate sRNAs were located on the more conserved replicon 1, all of which were conserved within representatives of the Bcc, the *B. pseudomallei* group and the *B. xenovorans* group. Three candidate sRNAs were located on replicon 2, these were less conserved and occurred only within the Bcc and the *B. pseudomallei* group (Table S2). Most candidate sRNAs were more conserved than directly adjacent intergenic sequences (Fig. 1, Fig. S1).

**Figure 5:**
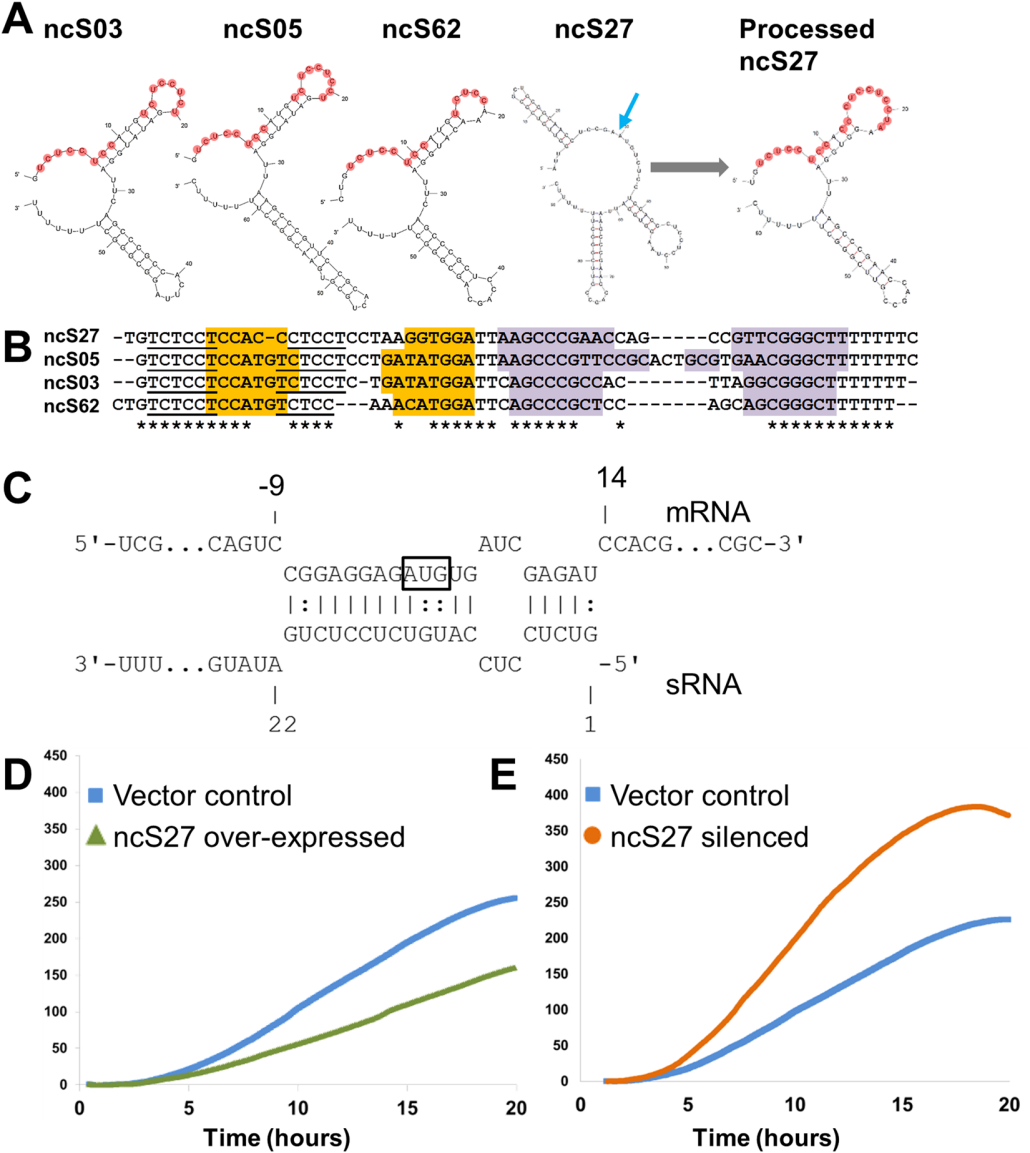
Homologous double-hairpin RNAs. (**A**) Secondary structures of four homologous sRNAs with Rfam accession RF02278 (“toxic small RNAs”, named so for their ability to inhibit growth in *E. coli* ^62^). dRNA-Seq data indicates a processing site for ncS27 (blue arrow), resulting in a shorter form with a secondary structure more similar to the other three homologues. CU-rich regions are highlighted in red. (**B**) Alignment of sRNAs (includes the processed form of ncS27). Paired bases are highlighted (orange: first hairpin, purple: second hairpin). Underlined: reverse complement of ribosome binding sites. (**C**) RNA duplex between ncS03 and BCAL3043 (6-phosphonolactonase). Both CU-rich regions of ncS03 are involved in duplex formation. mRNA nucleotide positions are relative to start codon (boxed). (**D**) Overexpressing ncS27 attenuates growth. (**E**) Silencing of ncS27 increases growth rate. The *in situ* cell density was measured by proxy of backscattered light and is expressed in arbitrary units. The expression level of the overexpression and silencing constructs exceeded the expression level of the native ncS27 by >60 fold in exponential growth phase.

ncS03, ncS05, ncS27 and ncS62 are homologous sequences (Fig. 5), three of which are very short, consisting almost entirely of two hairpin stem loops. The fourth homologue, ncS27, can be processed into an equally short form. We designated these sRNAs as “double-hairpin RNAs”. Three of these homologues also possess a common upstream motif (Fig. S3), while none of the remaining 11 candidate sRNAs share upstream sequence similarity. Genomes of other *Burkholderia* sp. all contain three to five homologous double-hairpin RNAs. Seven candidates, including the double hairpin sRNAs, contain one or more CU-rich stretches in unpaired regions of their secondary structure (Figs 4 and 5).

The 5′-end of transcripts is very precisely mapped by dRNA-Seq.^19^, whereas the 3′-end of RNAs can be identified by an abrupt decrease in coverage at a distinct location only if the transcript is very short and strongly transcribed. Such 3′-ends can only be distinguished from over-represented 5′-ends if the abrupt decrease in coverage occurs at distances from TSS other than the trimmed read length (95 nt). This was the case for 13 of the 15 selected sRNAs. To verify these 3′-ends, and to establish the 3′-ends of candidates not clearly defined in dRNA-Seq data, RACE was performed for eight candidates (Table 1, Doc. S1). Sequenced 3′RACE fragments confirmed dRNA-Seq data and ended downstream of the rho-independent terminator, including the U-rich stretch. ncS06 was shorter than initially predicted, ending at a U-rich stretch which was not preceded by a hairpin structure. The sequence of all candidate sRNAs ended in at least 4, and up to 9 transcribed Us.

**Figure 6:**
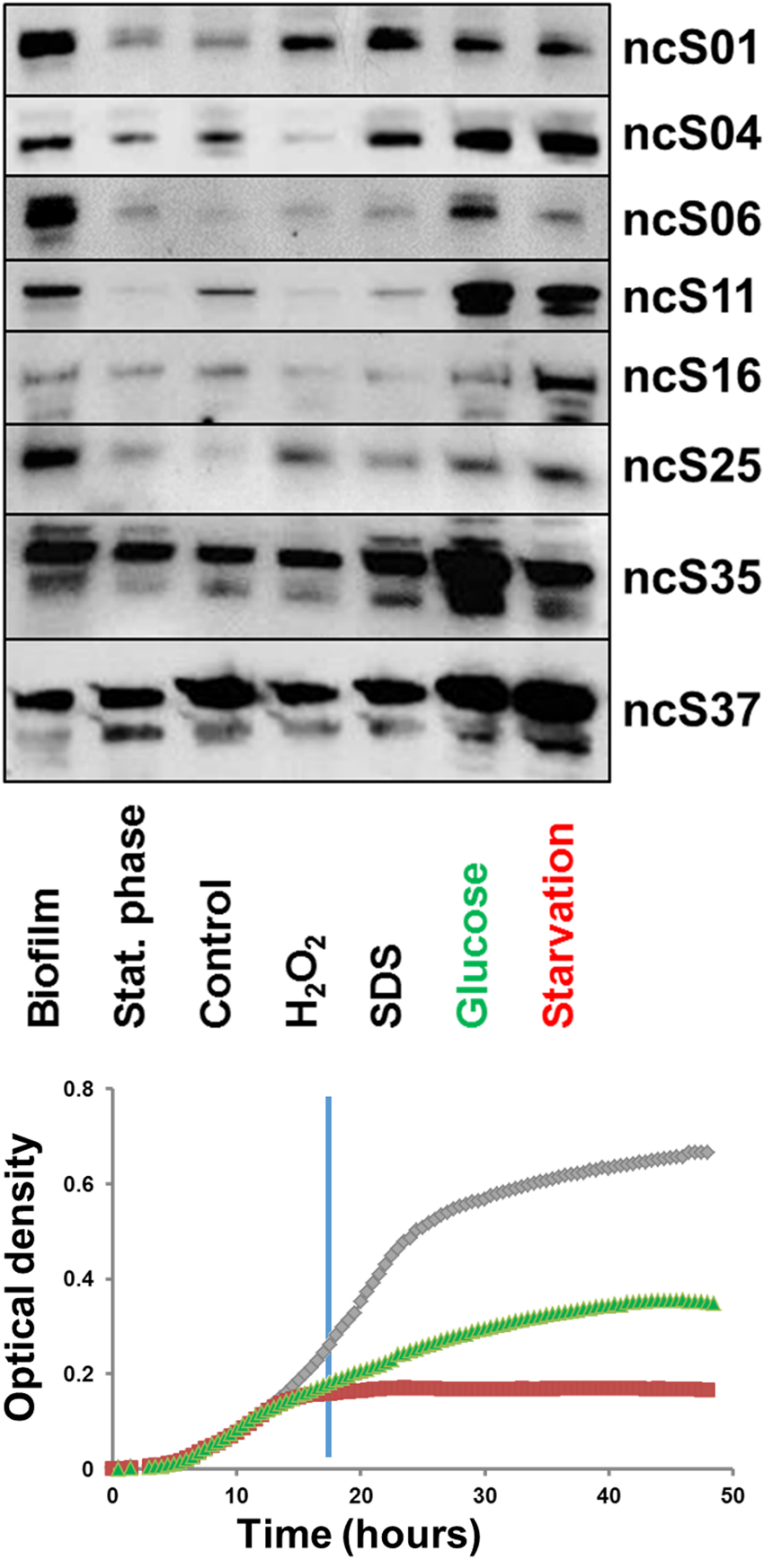
Expression of sRNAs. Northern blots revealed increased expression of many sRNAs in biofilms and in mineral medium, compared to planktonic control in LB (upper panels). Images are representative of two to three biological replicates. Full size Northern blot images of all replicates are presented in Fig. S6. Panels were cropped from membrane 1 (ncS37), membrane 4 (ncS04, ncS06, ncS16), membrane 6 (ncS01, ncS11) and membrane 8 (ncS25, ncS35). Lower panel: Representative growth curves in media used for conditions control (grey), glucose medium (green) and starvation (red) to illustrate differences in growth rate (lower panel) at time of harvest (blue line). Growth curves were measured in microtiter plates, with intermittent shaking.

Northern Blotting of ten sRNAs largely confirmed size predictions from dRNA-Seq and 3′RACE. An exception was ncS06 which appeared much shorter than predicted by 3′RACE (Fig. 4), indicating processing. The probe used for Northern blotting anneals upstream, and the 3′RACE primer downstream of the processing site (Doc. S2). The probe for ncS25 consistently produced two bands, only the shorter band changed intensity across conditions (Fig. 6) and could be assigned to ncS25 by analysis of a deletion mutant (Fig. 4). Some sRNAs consistently produced more than one band, which can be attributed to processing (Doc. S2) or alternative TSS (ncS54, Fig. S1).

### Expression profiles of candidate regulatory sRNAs

Expression of 14 sRNAs from this study was analysed by qPCR. Twelve sRNAs were significantly higher expressed in biofilms than in log phase planktonic cultures (Table 2). Expression was also higher in stationary phase than in exponential phase for seven of these sRNAs. To assess whether quorum sensing (QS) was involved in this upregulation, we investigated sRNA expression in *B. cenocepacia* J2315 deletion mutant strains with either the one or two homoserine lactone synthases inactivated (Δ*cepI* andΔ*cepI*Δ*cciI*, respectively)^27^. However, expression of sRNAs compared to wild type was not altered in these mutant strains (data not shown).

**Table 2:** Fold changes in sRNA expression determined by qPCR.

	BF	Stat. phase	Starvation	Glucose medium	Osmotic stress	Low pH	Low iron
ncS01	3.7	—	2.7	—	—	−3.9	—
ncS02	1.7	3.1	—	—	2.6	—	—
ncS03	2.9	—	2.6	8.0	2.7	3.5	—
ncS04	—	—	—	—	—	—	—
ncS05	3.5	2.2	3.9	6.9	2.9	—	—
ncS06	6.3	3.4	9.5	3.6	—	—	—
ncS11	34	13.9	6.0	—	—	—	—
ncS16	6.1	3.9	6.6	—	—	—	—
ncS25	8.3		2.8	2.1	—	—	—
ncS27	9.7	2.4	3.9	4.3	—	—	—
ncS35	7.8	3.0	3.2	3.5	—	—	—
ncS37	—	—	2.6	—	—	—	—
ncS54	3.1	—	2.0	—	—	—	—
ncS63	6.4	−26	−8.1	−8.5	—	−7.3	53
Hfq1	—	—	3.4	2.7	n/a	n/a	n/a
Hfq2	1.6	1.5	—	—	n/a	n/a	n/a

Fold changes were calculated in comparison to planktonic cultures in LB medium in exponential growth phase. Thresholds: p ≤ 0.05 and ≥1.5-fold change. BF: Biofilm, n/a: not analysed, -: expression change not significant.

Cultures in exponential phase were exposed to low iron (dipyridyl), membrane stress (SDS), oxidative stress (H_2_O_2_), osmotic stress (NaCl) or low pH for 20 min before harvesting for qPCR, to determine if sRNAs expression could be triggered by stress, as has been shown for other sRNAs^28^. Largely, this short time exposure did not result in significant induction of sRNA expression, although some sRNAs were slightly up-regulated by osmotic or pH stress (Table 2). ncS63 showed a significant and high-fold up-regulation in response to low iron. Its 5′-adjacent gene BCAL2297 is up-regulated to the same fold change, Cq values indicate an overall much lower expression of BCAL2297 in comparison with ncS63, corresponding to their coverage data (Fig. S1, Table S4).

In contrast to the prevailing lack of induction by stress, growth in nutrient depleted medium resulted in increased levels of 12 sRNAs (Table 2), five of these sRNAs were also more abundant in glucose medium. These findings could largely be confirmed by Northern blotting (Fig. 6). Hfq1 was also up-regulated in nutrient depleted medium, and Hfq2 in stationary phase and biofilms (Table 2). Previously, it was already shown that Hfq1 and Hfq2 do not change expression in several stress conditions, e.g. oxidative stress, low pH and low iron^29^.

### Computational target prediction for candidate sRNAs

Putative targets for candidate sRNAs were predicted with CopraRNA, an algorithm which takes accessibility of interaction sites and conservation of putative targets into account. Only regions surrounding the start codon of annotated genes, from 200 nt upstream to 100 nt downstream of the start codon were considered for target prediction, because most confirmed sRNA-mRNA interactions are located in those regions. To evaluate the predictions obtained with the CopraRNA algorithm, they were compared with predictions by RNApredator. RNApredator also takes accessibility of interaction sites into account, but scans the whole CDS plus 200 nt directly up- and downstream of the CDS. In the majority of cases, RNApredator predicted interactions in the same location as CopraRNA (Table S5). The proportion of matching locations was particularly high for the double-hairpin RNAs (>80%).

Target genes predicted by CopraRNA are mainly involved in carbon compound transport and metabolism, transcriptional regulators and cell envelope components such as outer membrane proteins and porins; different functional categories over-represented among targets vary between different sRNAs (Fig. S4, Table S5, Doc. S2). Only one sigma factor, extracytoplasmic function (ECF) sigma factor BCAL1947, and no genes involved in QS were present on the target lists. Many mRNAs were present on multiple target lists. A transcriptional regulator (BCAL1948) directly adjacent to above mentioned ECF sigma factor was present on nine sRNA target lists. In particular, ncS11 has extensive sequence complementarity to the coding region of this gene (Doc. S3).

Most predicted targets of double-hairpin RNA fall into categories metabolism and transport of amino acids, carbohydrates and aromatic compounds, e.g. gluconate permease (BCAL3365), galactonate transporter (BCAL0184, BCAM2500), glycerol kinase (BCAL0925), glycerol-phosphate transporter (BCAL3041), 6-phosphogluconolactonase (BCAL3043) and salicylate hydroxylase (BCAM1274). Predicted targets of ncS16 include a higher percentage of outer membrane and cell envelope components, as well as genes for transport of inorganic compounds. The predicted targets of ncS63 include a higher percentage of genes encoding proteins involved in energy production, e.g. succinate dehydrogenase (*sdhA*, BCAM0969), NADH dehydrogenase subunit B (*nuoB*, BCAL2343) and cytochrome O ubiquinol oxidase (BCAL2141). Predicted targets of ncS63 are also involved in detoxification of reactive oxygen compounds, e.g. catalase (*katB*, BCAL3299) and alkyl hydroperoxide reductase subunit F (*ahpF*, BCAM1216).

Functional categories significantly over-represented were organic acid transport (ncS03, ncS05), glycolysis (ncS62), outer membrane and cell envelope components (ncS16, ncS37), peptidases (ncS06), signal peptides (ncS02, ncS03, ncS37), iron binding (ncS63) and respiration (ncS16, ncS63). For sRNAs with CU-rich sequences in un-paired regions, a high proportion of predicted interactions was located within or overlapping the region 20 nt upstream and downstream of the start of annotated gene starts, with the CU-rich sequence covering the the ribosome binding site (RBS). This is in particular evident for the double-hairpin RNAs, for which 55–80% of predicted interactions fall in this region.

### Effect of overexpression and silencing of double-hairpin RNA ncS27

To overexpress the double-hairpin RNA ncS27, the entire sequence of ncS27 was cloned into an expression vector, under the control of an inducible promoter. To silence ncS27, the 5′ end of its sequence, containing the CU-rich region predicted to interact with RBS regions of target genes, was cloned into the same vector in reverse orientation. Overexpression and silencing of ncS27 was investigated in planktonic cells and in biofilms. In planktonic cultures, overexpression of ncS27 attenuated growth (Fig. 5D), whereas silencing of ncS27 had the opposite effect (Fig. 5E). Similarly, respiration rates were attenuated in the overexpression mutant, and enhanced in the silencing mutant (Fig. S5). In biofilms, overexpression or silencing of ncS27 had no significant effect on cell counts and on respiration rate (data not shown).

## Discussion

In the present study, 123 short transcripts expressed in *B. cenocepacia* J2315 biofilms have been identified and classified into two groups, independently transcribed sRNAs and short 5′UTRs.

Known regulatory sRNAs of other bacterial species tend to be more conserved than the rest of the intergenic region they are encoded in^30^, and they tend to have a low z-score^31^. sRNAs often function by binding to the RBS, chaperoned by Hfq, occluding it and thus reducing translation rate; a long stretch of transcribed Us is necessary for sRNA-interaction with Hfq^32^. Candidate sRNAs of *B. cenocepacia* show relatively high local conservation and structural significance, and their terminators are followed by a stretch of four to nine Us. The reverse complement of RBS in un-paired regions of some sRNAs makes it possible they act via RBS occlusion; binding to Hfq has been shown for ncS04^17^. Together, these observations substantiate that sRNAs identified in the present study are plausible candidates for functional sRNAs.

Interestingly, the average GC-content of all identified sRNAs is with 58.8% lower than genome average and also lower than the average for IGRs (63% GC)^18^ for *B. cenocepacia* J2315. Structural RNAs (16s rRNA 55.2% GC, 5S rRNA 50.4% GC) and essential small non-coding RNAs (tmRNA 57.3% GC, 6S RNA 56.5% GC) also have a GC-content lower than IGR average. Base composition of RNAs can differ from the rest of the genome in Archaea, and GC-content bias has been used as criterion for sRNA prediction in these cases^33^. The GC-content bias observed for sRNAs identified in *B. cenocepacia* could therefore be a further indication that these sRNAs are functional.

Many short 5′UTRs presented in this study likely have a cis-regulatory function. Genes adjacent (3′ end) to the short 5′UTRs identified in *B. cenocepacia* include homologues of genes known to harbour cis-regulatory structures in other bacterial species, e.g. ribonuclease E^34^ (BCAL2888), a proline-betaine transporter^35^ (BCAL1252), ribosomal proteins^36^ (BCAL0115, BCAL2091, BCAL2765, BCAL2714, BCAL3348), *carA* ^37^ (BCAL1260) and tRNA-synthetases^36^ (BCAL3373, BCAL3436). Attenuation, observed for many short 5′UTRs, is indicative of a cis-regulatory function. Overall, 5′UTRs of *B. cenocepacia* are an abundant source of short RNAs, and cis-regulatory 5′UTRs seem to be as numerous as in many other bacterial species^38^. Whether the short 5′UTRs identified in the present study have a further regulatory function *in trans*, as shown for the *irvA* gene of *Streptococcus mutans* ^39^, is at present unknown.

To obtain a first indication about the possible roles of candidate regulatory sRNAs, computational target prediction was performed. However, the lists of computationally predicted targets are expected to contain a large proportion of false positives, and all targets still need to be experimentally verified. Moreover, it is possible that candidate sRNAs act by a different mechanism which does not involve sequence complementarity to a target; such interactions cannot be detected by computational target prediction.

The high-fold up-regulation of ncS63 specifically in iron-depleted medium, its genome context and predicted target profile suggest that ncS63 could participate in the cellular response to iron depletion and in maintaining iron homeostasis. ncS63 is-co-transcribed with BCAL2297, encoding hemin-uptake protein HemP. *HemP* features an upstream Fur motif^40^, which places ncS63 under the regulatory control of the Fur repressor. Many predicted target genes of ncS63 encode for proteins containing iron in the form of heme or iron-sulfur clusters. ncS63 thereby shares characteristics with RyhB, a small RNA under Fur-repression in *E. coli* ^41^. RyhB regulates *sdhA* and other genes involved in respiration, and superoxide dismutase (*sodB*), while ncS63 targets include *sdhA* and *katB*. In addition, genes for iron-containing proteins are enriched in the target lists of RyhB^42^. ncS63 could therefore be a functional analogue of RyhB, although genome context and secondary structure are different.

The double-hairpin RNAs bear structural similarities to NmsRs, two homologous sRNAs occurring as tandems in the genome of *Neisseria meningitidis*, a pathogenic beta-proteobacterium^43^. Both NmsRs contain two CU-rich stretches which are located in unpaired regions of their secondary structure, and which show complementarity to the RBS regions of genes of the tricarbolic acid (TCA) cycle; these genes were also up-regulated in a NmsR double-deletion mutant^43^. Double-hairpin RNAs occur not in tandem, their up to five homologues are distributed throughout *Burkholderia* genomes. Similarly, the QS-regulatory sRNAs (Qrrs) of *Vibiro* sp. occur in up to five distinctly located homologues. Qrrs regulate QS in a base-pairing-dependent manner^44^, and Qrr1 is located directly adjacent to *luxO*. ncS62 is located in close proximity to *cepI*, the major homoserine lactone synthase in *Burkholderia* sp. Lists of computationally predicted targets for double-hairpin RNAs suggest that they could play a role in regulating transport and metabolism of carbon compounds, in particular of carbohydrates, but sequence complementarity to genes in the TCA cycle or to QS genes was not found.

Most candidate sRNAs were more abundant in conditions with growth arrest and low amino acid availability; factors which also occur in biofilms. Accumulation of sRNAs under nutrient stress, notably amino acid starvation and stringent response, has been described for known sRNAs like Spot 42^45^ and increased levels of sRNAs in stationary phase cultures are frequently observed^46^. Increased abundance of sRNA transcripts can be the result of transcription induction as well as relative increase due to protection from degradation, mediated by secondary structure and/or or binding to Hfq. Promoter sequences as well as RNA degradation pathways of *B. cenocepacia* remain largely uncharacterised. What regulates the observed accumulation of sRNAs in this bacterium is thus yet unknown. Interestingly, the double-hairpin RNAs, predicted to regulate carbohydrate uptake and metabolism, are more abundant in a glucose medium than under starvation, although growth rate was higher in glucose medium than under starvation at point of harvest. It suggests that these sRNAs could have a role in maintaining glucose homeostasis under conditions of amino acid starvation and in reducing phosphosugar stress^47^. On the other hand, in a medium rich in amino acids overexpression of the double-hairpin RNA ncS27 attenuates growth, which seems to confirm a role in repressing amino acid uptake and metabolism. Expressing the reverse complement of the CU-rich region had the inverse effect of overexpression, which seems to confirm that specifically this region is exerting the regulatory effect of ncS27. A specific role for double-hairpin RNA ncS27 in biofilm formation was not apparent from our results.

Several sRNAs have sequence complementarity with a gene encoding a regulatory protein (BCAL1948), in particular ncS11, with sequence complementarity stretching over 40 nt. ncS11 is strongly up-regulated in stationary phase and biofilms, BCAL1948 is downregulated under these conditions^29^. The adjacent ECF sigma factor (BCAL1947) is itself a computationally predicted target of sRNAs. This ECF sigma factor is up-regulated in stationary phase, growth under oxygen limiting conditions and in the presence of hydrogen peroxide, conditions which all cause growth arrest^29^. Whether ncS11 regulates expression of BCAL1948, and whether BCAL1948 regulates expression of the ECF sigma factor is at present unknown. Further studies are under way to confirm sRNA targets and to investigate involvement of sRNAs in regulatory circuits of *B. cenocepacia*.

## Methods

### Bacterial strains and culturing conditions

*Burkholderia cenocepacia* J2325 (LMG 16555) was routinely maintained on LB agar (Oxoid). Experiments were performed in LB broth, pH 7, or a phosphate buffered mineral medium^29^, pH 7, supplemented with yeast extract and peptone or with glucose as required (see below).

Biofilms were grown in LB broth in 96 well microtiter plates and harvested as described before^48^. Planktonic cells were grown in glass flasks in a shaking incubator at 150 rpm, snap cooled in ice water and harvested by centrifugation at a fixed density of 5 × 10^8^ (log phase cultures) unless otherwise noted. For stress conditions the following compounds were added to log phase cultures in LB medium 20 min prior to harvesting: 0.4 mM 2,2′-dipyridyl (Sigma-Aldrich) for reduced iron availability, 1 M HCl to lower the pH to 5.0, 0.05% H_2_O_2_ for oxidative stress, an additional 2% NaCl for osmotic stress, and 0.01% sodium dodecyl sulphate (SDS) for membrane stress. Mineral medium supplemented with 0.06% yeast extract and 0.12% peptone (Bacto peptone, Becton Dickinson) was used to induce growth arrest (to mimic starvation), while mineral medium supplemented with 0.03% yeast extract and 0.03% peptone and 10 mM glucose was used as “glucose medium”.

Late log phase cultures were harvested at a density of 1 × 10^9^ colony forming units (CFU) per ml and stationary phase cultures at a density of 2 × 10^9^ CFU/ml. For condition “starvation”, cells were harvested at 4 × 10^8^ CFU^/^ml, approx. 1 hour after their optical density had stopped increasing.

Unmarked deletion mutants for the major QS systems of *B. cenocepacia* (Δ*cepI*, Δ*cepI*ΔΔ*cciI*) were obtained from Silvia Buroni, Pavia, Italy^27^, an unmarked deletion mutant of ncS25 was constructed by allelic replacement^49^ (Table S6).

ncS27 was selected for overexpression and silencing, because it is processed according to dRNA-Seq data (Fig. S1). The processing would remove additional bases from the 5′end introduced by the cloning procedure. An existing vector containing a system for rhamnose-inducible protein expression, pSCrhaB2^50^, was modified for RNA expression; RBS and start codons were removed via inverse PCR. For overexpression, ncS27 was amplified from 3 nt upstream of the start codon until 12 nt downstream of its terminator. For silencing, the reverse complement of the 5′ end of ncS27 was cloned in reverse orientation into the multiple cloning site. Primers used for vector construction and cloning are listed in Table S6. The level of expression from the rhamnose inducible promoter was estimated by qPCR on cultures harvested in exponential growth phase at O.D._595_ 0.5.

Growth curves for overexpression and silencing mutants were monitored with a Cell Growth Quantifier (Aquila Biosystems), which measures the intensity of backscattered light every 30 s. Cells were grown in LB broth supplemented with 600 µg/ml trimethoprim and 0.2% rhamnose in a shaking incubator at 100 rpm. Respiration rates were measured in microtiter plates using a resazurin-based method (CellTiter-Blue, Promega)^51^. For planktonic growth, cell densities were normalised to 5 × 10^8^ CFU per well and fluorescence (λ_ex_: 560 nm and λ_em_:590 nm) was measured every 10 min in a plate reader (Envision, PerkinElmer). Cell counts in biofilms were determined by conventional plating.

### RNA-extraction

RNA was extracted using the RiboPure bacteria kit (Invitrogen), using the standard protocol, except that the proportion of ethanol added to crude extract prior to clean-up was increased to 1.25x to retain more small RNAs. Before DNase treatment, RNA was denatured by heating to 65 °C for 5 min and DNase incubation time was increased to 60 min. Total RNA quality and relative quantity of small RNAs in the extract was analysed with the Experion system (Bio-Rad) using StandSens chips.

### RNA-Sequencing and transcription start site annotation

Differential RNA-Sequencing (dRNA-Seq), global RNA-Sequencing (gRNA-Seq) and transcription start site (TSS) annotation was described previously^19^, using the most recent genome annotation^12^ with accession numbers AM747720, AM747721, AM747722, and AM747723. Array Express accession numbers for dRNA-Seq and gRNA-Seq data are E-MTAB-3381 and E-MTAB-2079.

### 3′ and 5′ RACE

5′RACE was performed as previously described^19^, using the 5′RACE System from Invitrogen. cDNA was generated from total RNA using a gene specific primer (GSP1) and then poly(C)-tailed using terminal deoxynucleotidyl transferase. cDNA was then amplified using a nested gene specific primer (GSP2) and the abridged anchor primer from the 5′RACE system.

For 3′RACE, total RNA was first poly(A)-tailed (Poly(A) Polymerase Tailing Kit, Epicentre). cDNA was generated using the 3´RACE System from Invitrogen, with an oligo d(T) adapter primer. cDNA was then amplified using a gene specific primer (GSP3) and the abridged universal amplification primer which anneals to the adapter part of the oligo d(T) primer from the kit. Where possible, PCR products were re-amplified with nested primers (GSP4). Primer sequences are listed in Table S6.

PCR products were cloned using the pGEM vector system (Promega). Between four and seven plasmid inserts were sequenced per analysis. Alignments of all RACE results are presented in Doc. S1.

### Computational analyses

Coverage of dRNA- and gRNA-Seq data was visualised with the Integrated Genome Browser^52^. Sequence conservation of intergenic regions were visualised using pre-computed whole-genome alignments of 8 genome-sequenced *Burkholderia* sp. of the Vista synteny viewer^53^ via the Integrated Microbial Genomes database^54^. Strains are listed in Fig. S1.

The mfold web server was used for RNA secondary structure prediction^55^. The Rfam database^56^ was used to search for homologues with known function. Rho-independent terminators were predicted with TransTermHP^57^, using the–all-context command line option to predict terminators independently from annotations. Sequence motifs in upstream sequences were searched for using MEME^58^. sRNA homolology searches were conducted using BLASTn^59^, with adjusted input parameters (word size: 7, Match/Mismatch Scores 1/−1, Gap existence cost -0, gap extension cost: 2) and with 65% query coverage and 60% sequence similarity cut-offs. Genome-sequenced strains included in BLASTn searches are listed in Table S2. Putative sRNA targets were predicted with CopraRNA^42^, and with RNApredator^60^. Input strains for CopraRNA are listed in Table S5. CopraRNA output is a list of 100 targets with the smallest p-values, present in at least 50% of input strains. The RNApredator output is a list of one interaction for each annotated gene, 7116 interactions for each sRNA. Functional enrichment analysis, based on the DAVID tool^61^, is incorporated in CopraRNA.

z-scores for MFE were calculated using 1000 randomly mononucleotide-shuffled sequences (CLC Genomics Workbench v. 8.5.1). The z-score is the number of standard deviations (σ) the MFE of the actual sequence (x) deviates from the mean MFE (μ) of shuffled sequences, (x − μ)/σ.

### Northern blots

Pre-cast 10% polyacrylamide TBE-urea gels (Bio-Rad) were loaded with 5 µg total RNA per lane. RNA samples were mixed with TBE-urea sample buffer (Bio-Rad) and denatured at 90 °C for 5 min. On each gel, two RNA ladders were run alongside: 0.1–2 kb RNA ladder (Invitrogen) and a small RNA marker (Abnova, fragment sizes 20 to 100 bases). Gels were run for 1 to 1.5 hours at 80 V. Before blotting, lanes containing RNA ladders were cut off the gel, stained with GelRed (Biotium), and the band position documented for later RNA size estimation.

RNA was blotted onto positively charged nylon membranes (Roche) in a Mini Trans-Blot Electrophoretic Transfer cell (Bio-Rad) for 1 hour at 300 mA, then cross-linked with UV-light. 5′DIG-labelled probes were obtained from Sigma. Membranes were hybridised at 42 °C overnight at 8 rpm, with 100 ng/ml DIG-labelled probes (Table S6) in ULTRA-Hyb-Oligo hybridisation buffer (Ambion). Membrane washing and immunological detection of probes was performed using the DIG Wash and Block Buffer Set and the DIG Luminescent Detection Kit (Roche). Luminescence was recorded with BioMax Light X-ray films (Carestream). Membranes were stripped using 0.5% SDS at 60 °C for one hour and re-used for hybridisation. Northern blots were conducted in triplicate, using RNA isolated from three independent biological replicates.

### Quantitative RT-PCR (qPCR)

cDNA was generated from 500 ng RNA using the qScript cDNA Synthesis kit (Quanta Biosciences). qPCR was performed in a Bio-Rad CFX96 Real-Time System C1000 Thermal Cycler using GoTaq qPCR Master Mix (Promega). Every sample was run in two technical replicates, and no-template- and no-RT controls were included for every primer pair. Cq values were normalised against two control genes with minimal expression changes across all tested conditions for data normalisation: BCAM0918, (encoding RNA polymerase D) and BCAL2367 (encoding a transcriptional regulator). Primer sequences are listed in Table S6.

Fold changes were calculated compared to a standard (mix of all cDNAs in experiment) and log-transformed. Normality of distribution was tested with a Kolmogorov-Smirnov test and changes in expression were analysed by One-Way ANOVA with a Tukey Post-hoc test using SPSS statistics v. 24 (IBM). Cq values are listed in Table S4.

### Data availability

All data generated or analysed during this study are included in this published article, as supplementary information files. Raw dRNA-Seq and gRNA-Seq data are available under Array Express accession numbers E-MTAB-3381 and E-MTAB-2079.

## Electronic supplementary material

Supplementary file 1 (Figures S1 to S6, documents S1 to S3)

Supplementary file 2 (Tables S1 to S6)
