# Supplementary material for: Identification of small RNAs abundant in Burkholderia cenocepacia biofilms reveal putative regulators with a potential role in carbon and iron metabolism
Source: Sci Rep. 2017 Nov 15;7:15665. doi: 10.1038/s41598-017-15818-3 (PMC5688073; doi:10.1038/s41598-017-15818-3)
Supplement: Supplementary file 1 — (Figures S1 to S6, documents S1 to S3) [file 41598_2017_15818_MOESM1_ESM.pdf]

Supplementary information for:

Identification of small RNAs abundant in *Burkholderia cenocepacia* biofilms reveal putative regulators with a role in carbon and iron metabolism.

Andrea Sass, Sanne Kiekens, Tom Coenye

Department of Pharmaceutical Microbiology, Ghent University, Ghent, Belgium
email: Tom.Coenye@UGent.be

Supplementary file 1:

Fig. S1. dRNA-Seq coverage data and conservation of 15 candidate sRNAs.

Fig. S2. Expression profiles of selected short transcripts and their adjacent genes.

Fig. S3. DNA Sequence motif found upstream of ncS03, ncS05 and ncS27.

Fig. S4. Functional annotation of computationally predicted sRNA targets.

Fig. S5. Respiration rates for ncS27 overexpression and silencing mutants.

Fig. S6. Full size images of Northern blots.

Doc. S1. sRNA sequences, with probe and processing sites indicated, and with secondary structures in Vienna format.

Doc. S2. Diagrams for selected sRNA-mRNA interactions.

Doc. S3. Alignments of RACE results.

Supplementary file 2 (separate): Multi-sheet Excel file.

Tab. S1. List of TSS in intergenic regions resulting in short transcripts.

Tab. S2: sRNAs with rho-independent terminator.

Tab. S3 List of short RNAs from 5'UTRs.

Tab. S4. qPCR Cq values.

Tab. S5. CopraRNA results for 15 candidate sRNAs.

Tab. S6: Probe and primer sequences.

Figure S1, part 1
sRNAs on chromosome 1

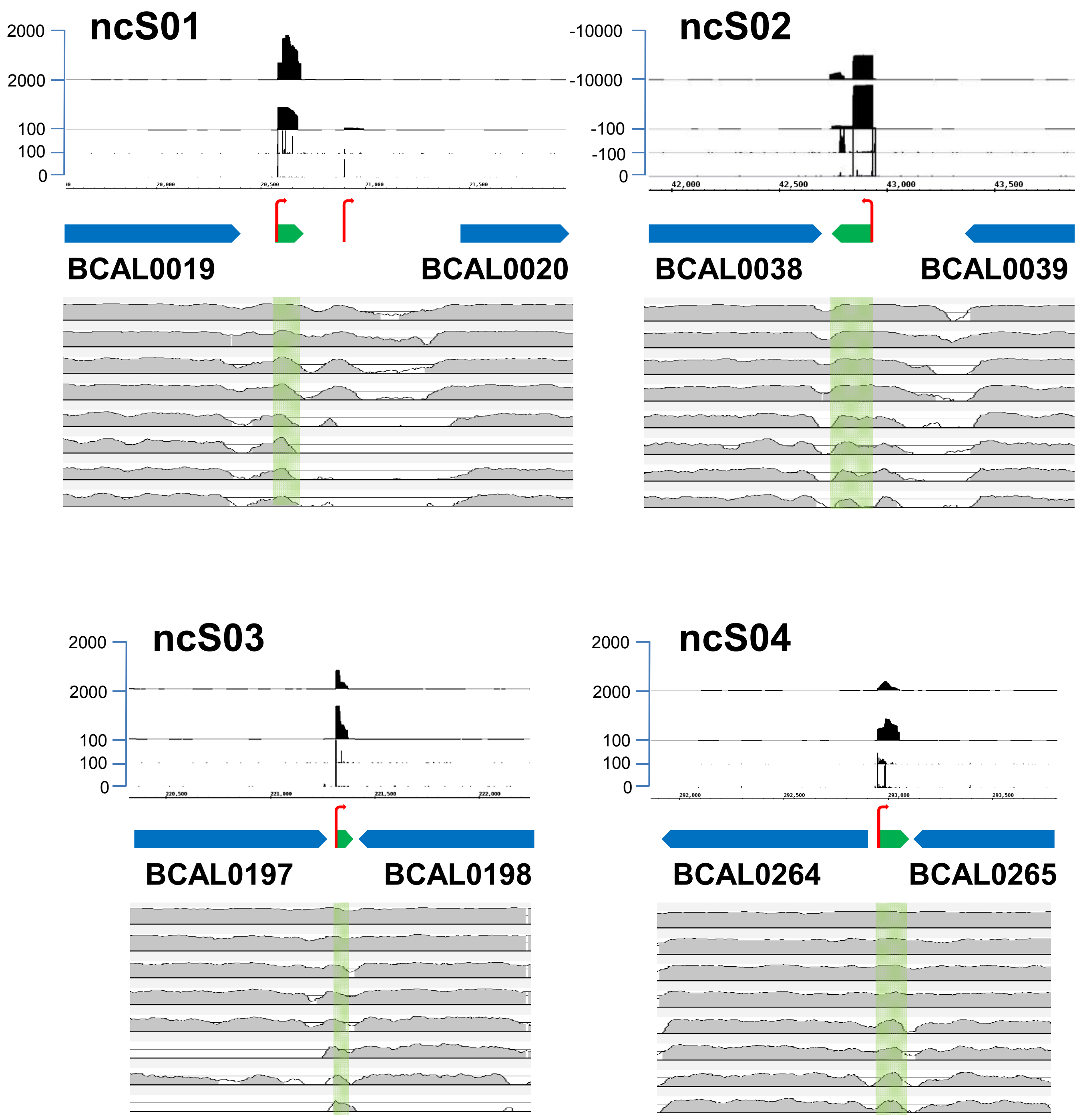

Figure S1, part 2
sRNAs on chromosome 1

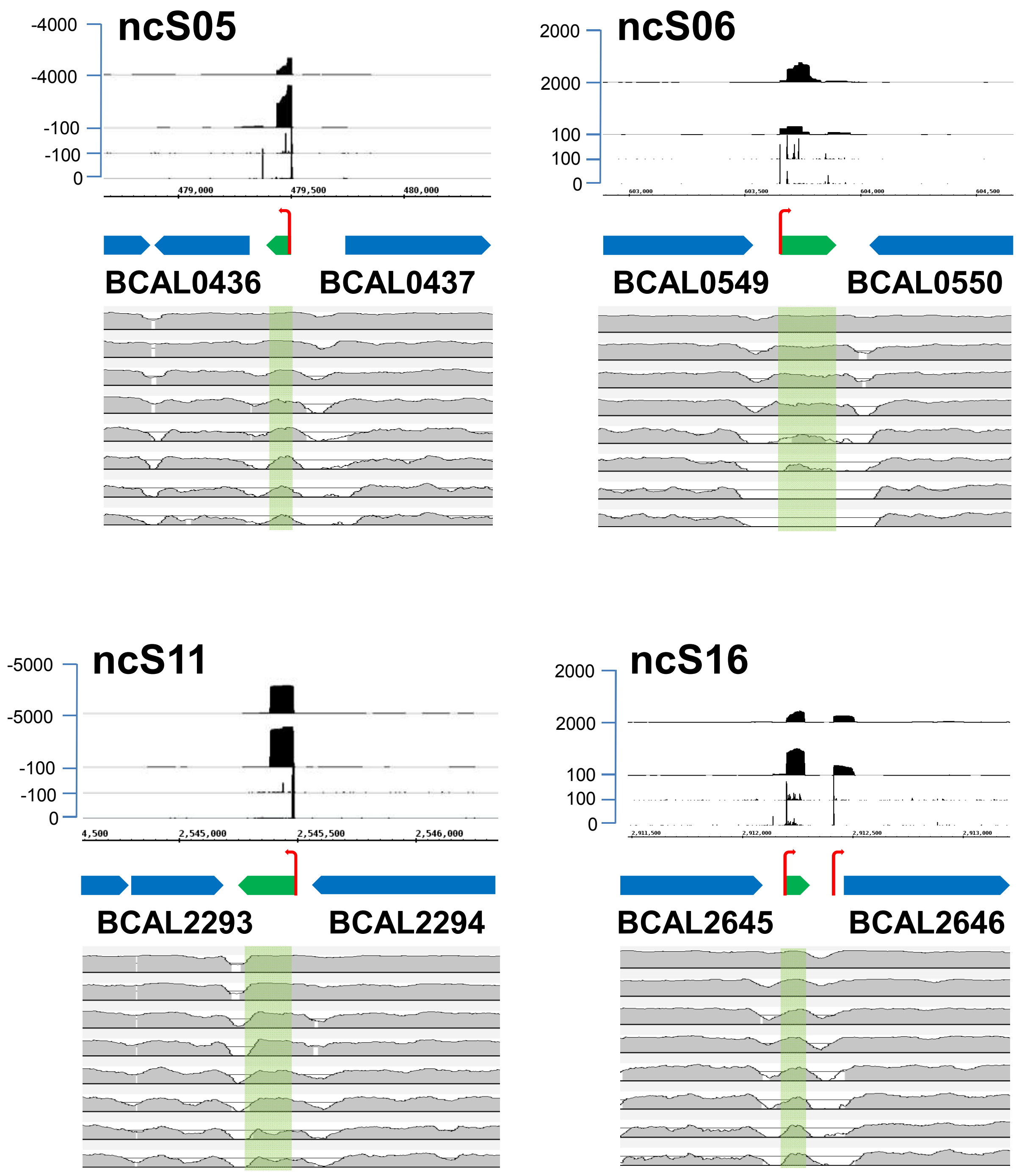

Figure S1, part 3
sRNAs on chromosome 1

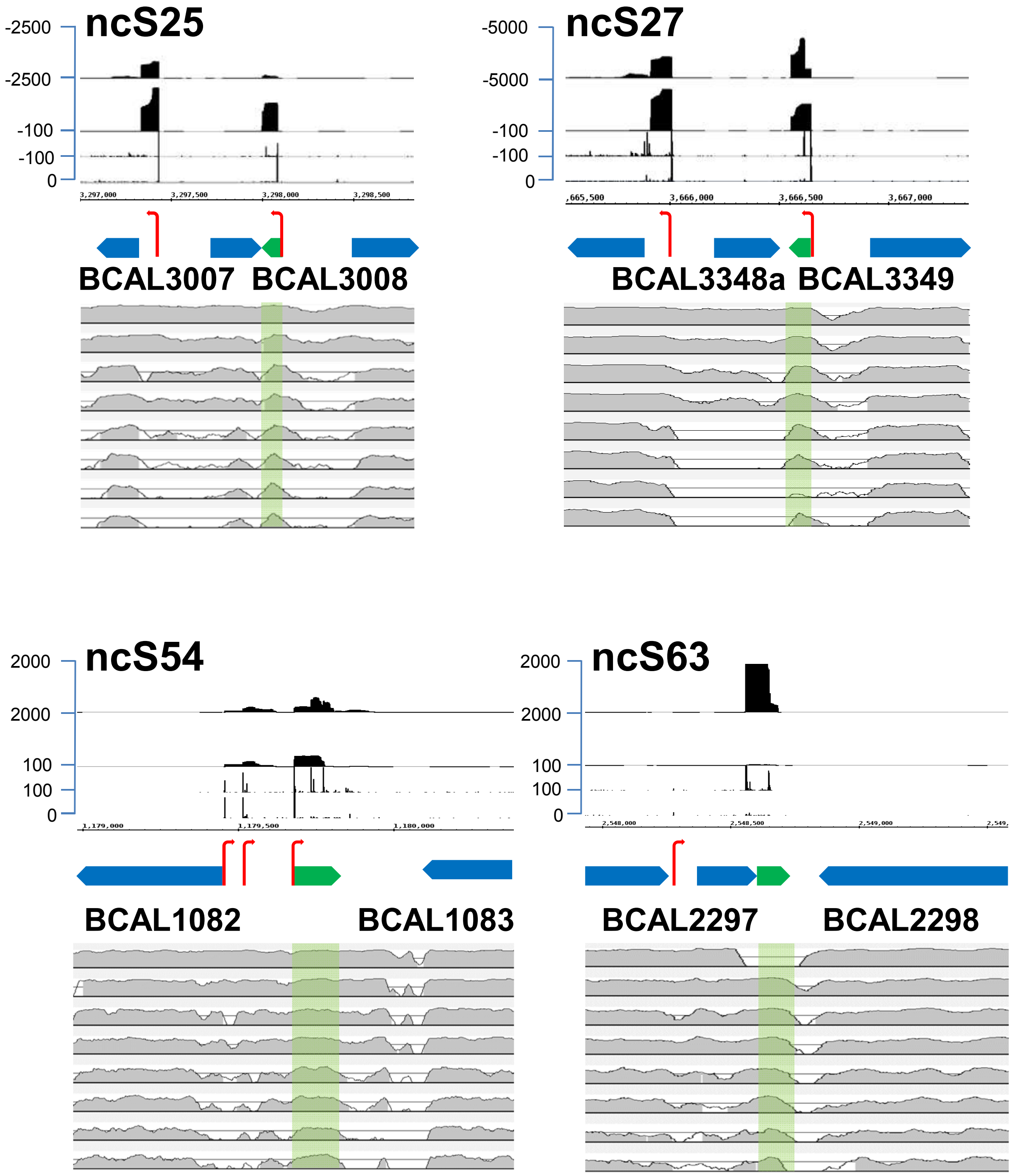

Figure S1, part 4
sRNAs on chromosome 2

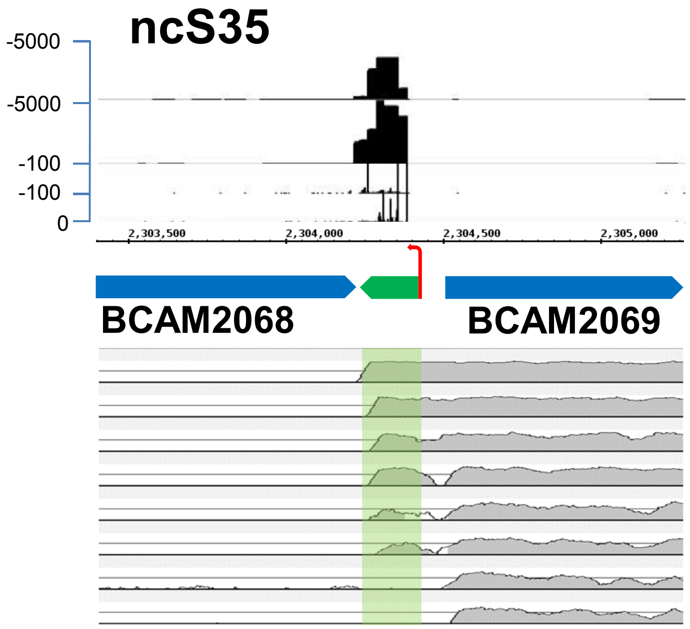

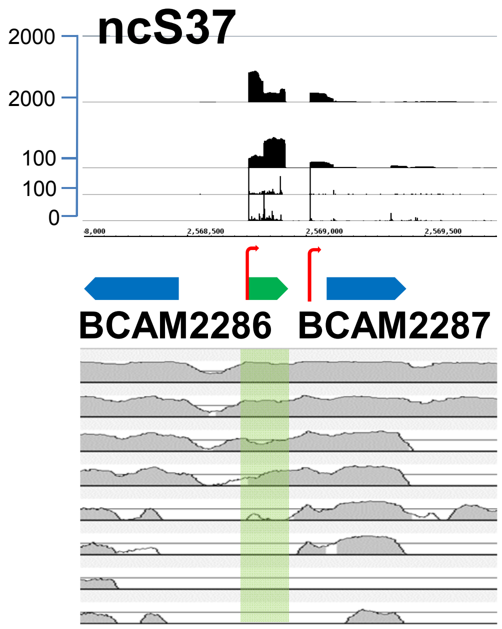

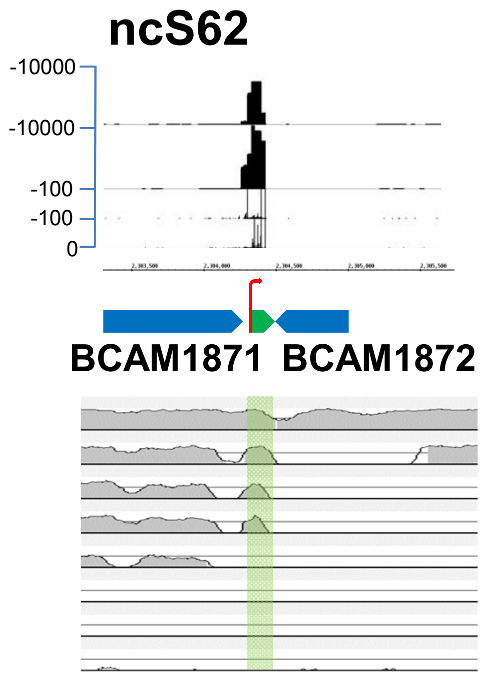

Figure S1

Upper panels, from top to bottom: **Coverage** (-TEX and +TEX) **and read starts per nucleotide** (-TEX and +TEX) **from dRNA-Seq data**. Red arrow: TSS. Green: sRNA. Blue: CDS.

Lower panels: **Conservation of sRNAs within IGRs.** Genome alignments visualised with Vista Point. Lines represent percentage similarity, similarities ≥ 70% are coloured in grey. Locations of sRNAs are shaded in green. Strains displayed for genome alignments, from top to bottom, with increasing phylogenetic distance: *B. cenocepacia* AU1054, *B. lata* sp.383, *B. vietnamiensis* G4, *B. multivorans* ATCC 17616, *B. pseudomallei* K96243, *B. glumae* BGR1, *B. phymatum* STM815, *B. xenovorans* LB400.

Figure S2

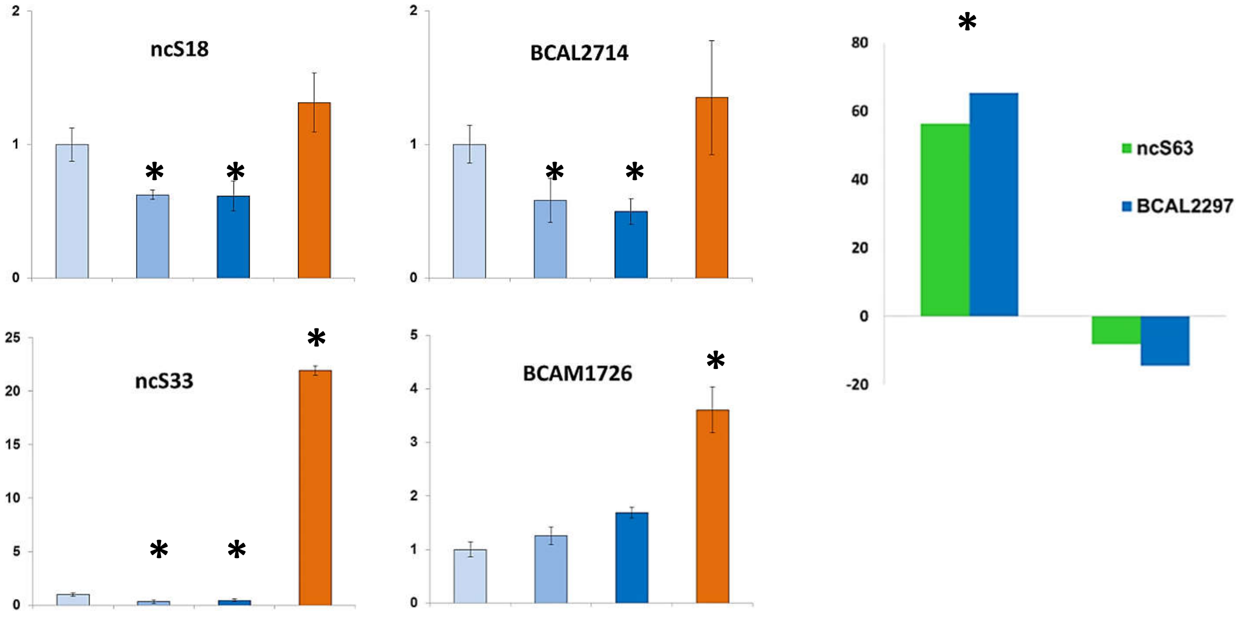

Figure S2: Expression of ncS18, ncS33 and ncS63 compared to their adjacent genes. Analysed by qPCR. Y-axis: Fold-changes normalised to exponential growth in LB. Asterisk: significant fold-changes compared to control ($p < 0.05$). (A) ncS18, but not ncS33 are co-regulated with its downstream genes. Conditions, from left to right: planktonic cultures (blue) in exponential phase, late exponential phase, stationary phase, and biofilm (orange). (B) ncS63 is co-regulated with its upstream gene. Conditions: LB with dipyridyl (left) and minimal medium which is supplemented with iron (right).

Figure S3

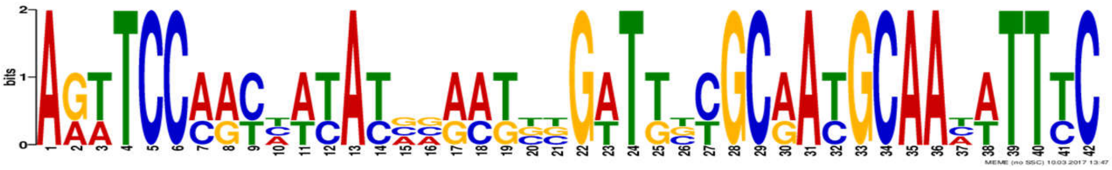

Figure S3: DNA sequence motif found upstream of ncS03, ncS05 and ncS27.

Figure S4

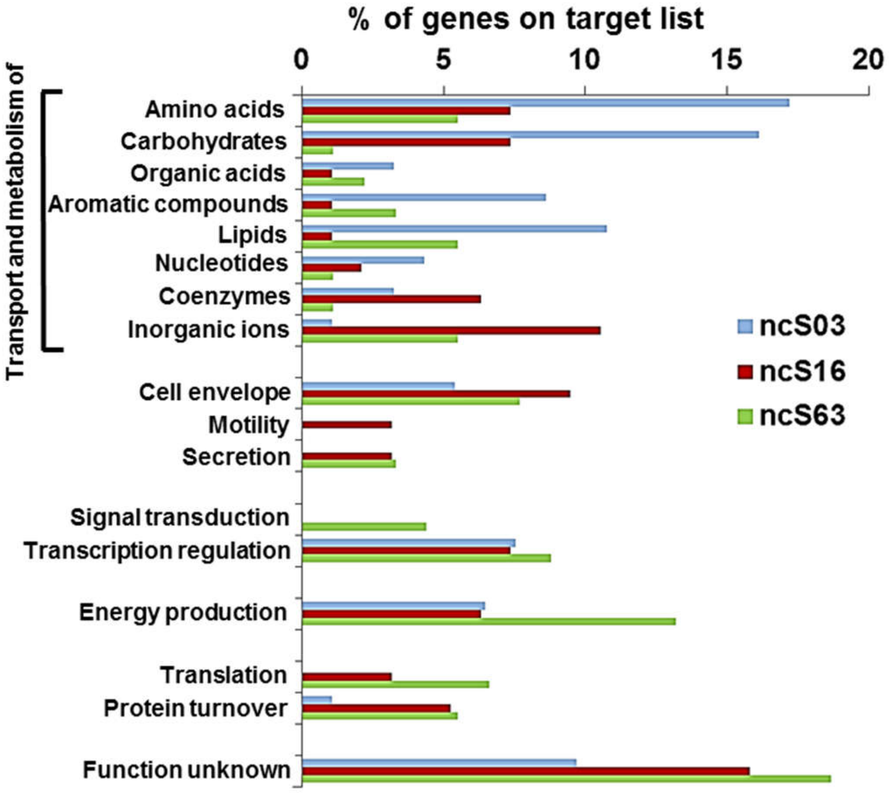

Figure S4: Functional annotation of computationally predicted sRNA targets. Targets for ncS03, a representative of the four homologous double-hairpin sRNAs, include mainly transport and metabolism of various carbon compounds. Targets of ncS16 are enriched in functional categories inorganic ion transport and metabolism and cell envelope components. Targets of ncS63 are enriched for genes involved in respiration.

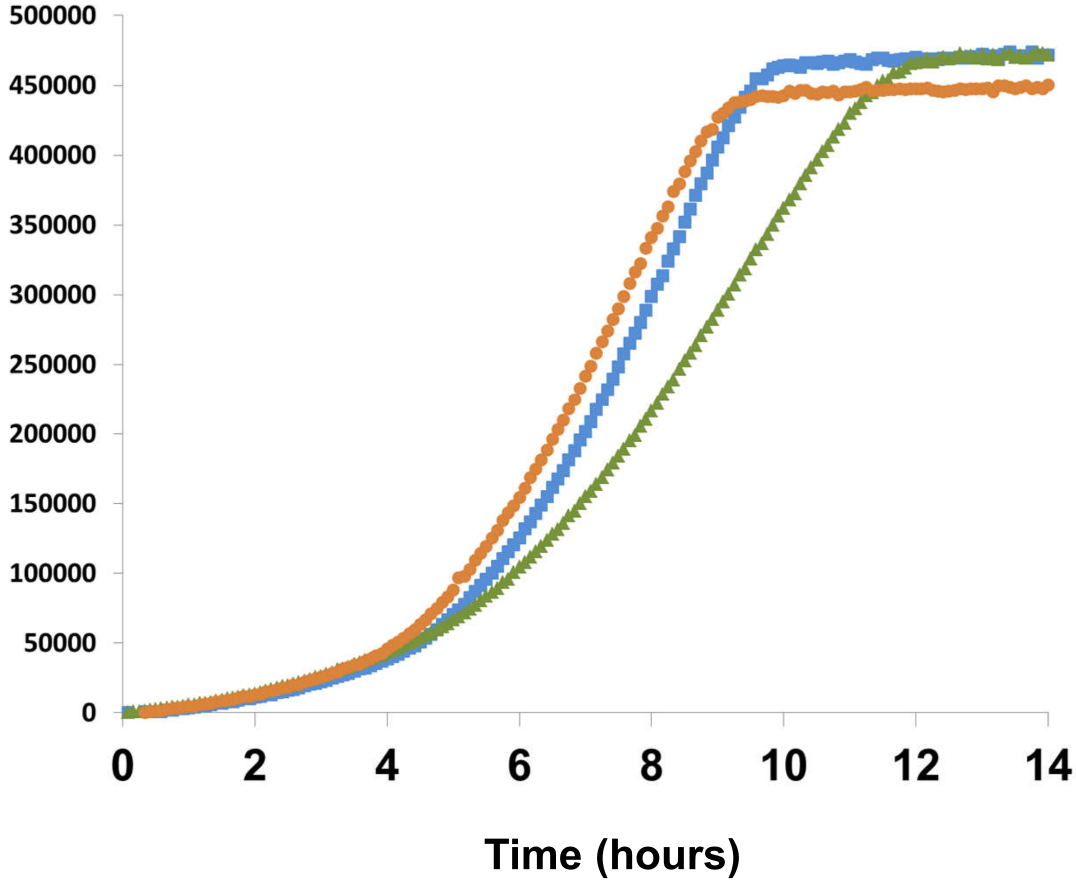

Fig. S5: Respiration rates for ncS27 overexpression and silencing mutants. Cell suspensions were mixed with CellTiter-Blue in microtiter plates and reduction of resazurin to pink and fluorescent resorufin was measured in a plate reader. Blue line: Vector control. Green line: Mutant overexpressing ncS27. Orange line: Mutant with ncS27 silenced. Fluorescence is expressed in arbitrary units and represents the mean of 5 wells.

Figure S6, part 1

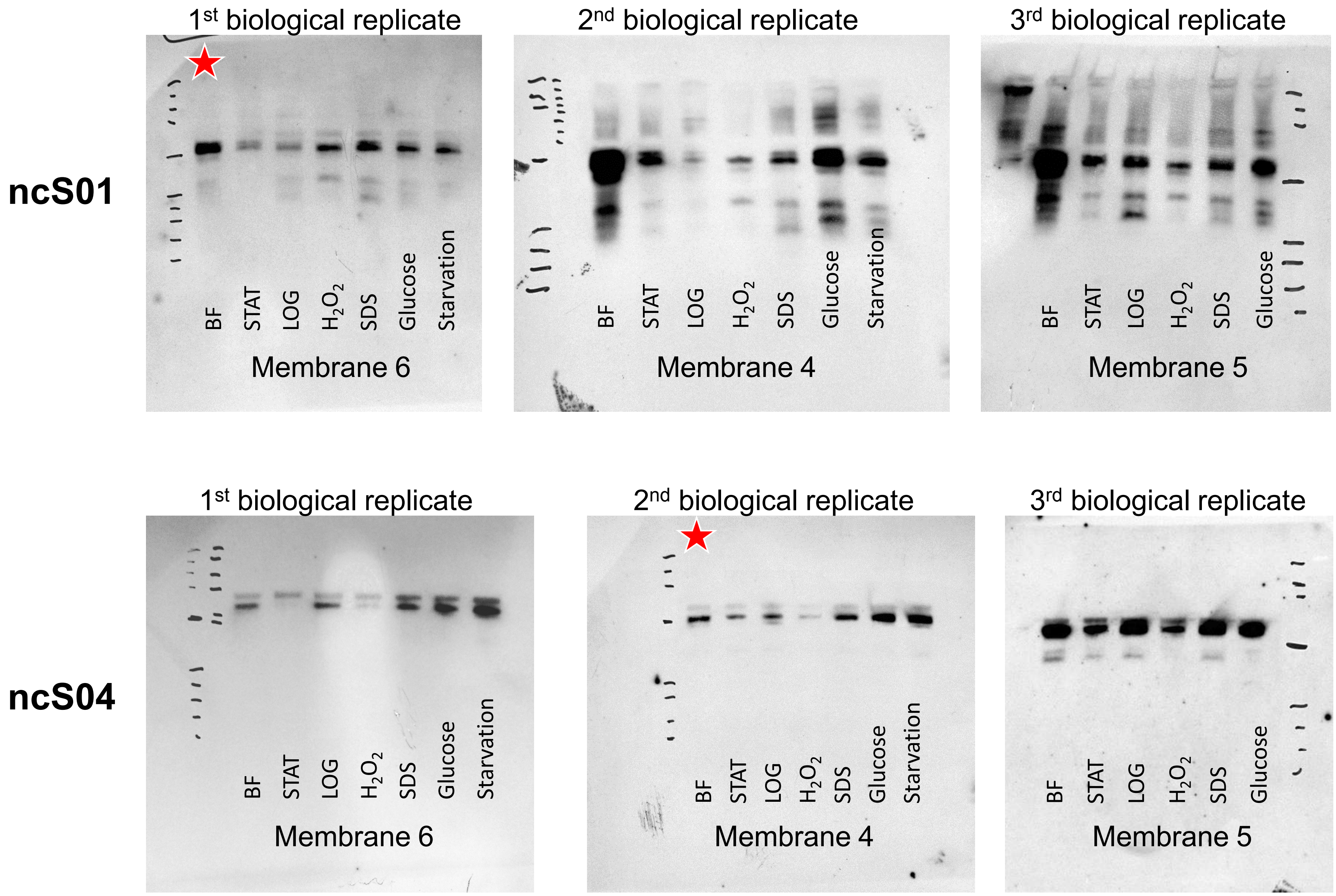

Figure S6, part 2

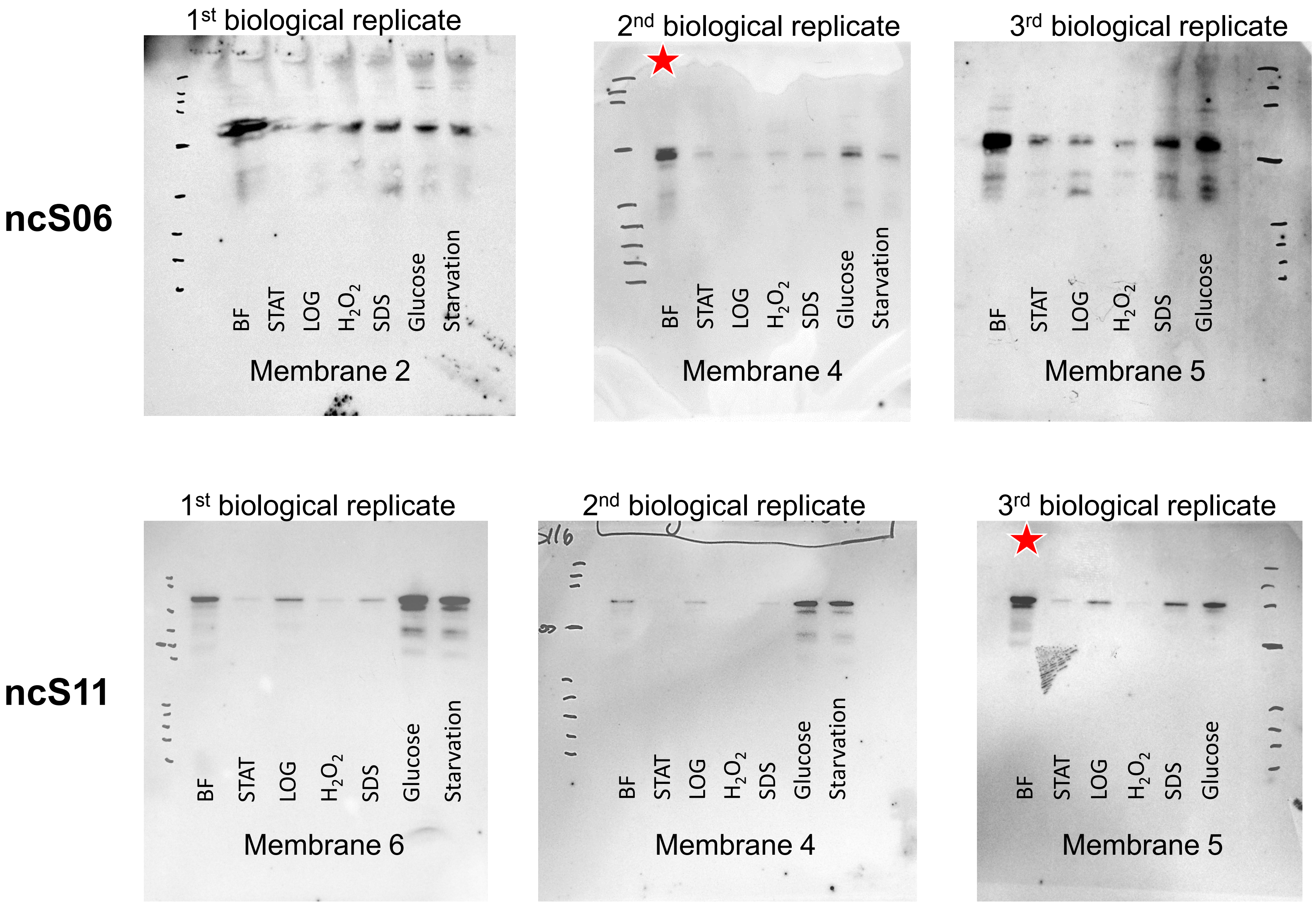

Figure S6, part 3

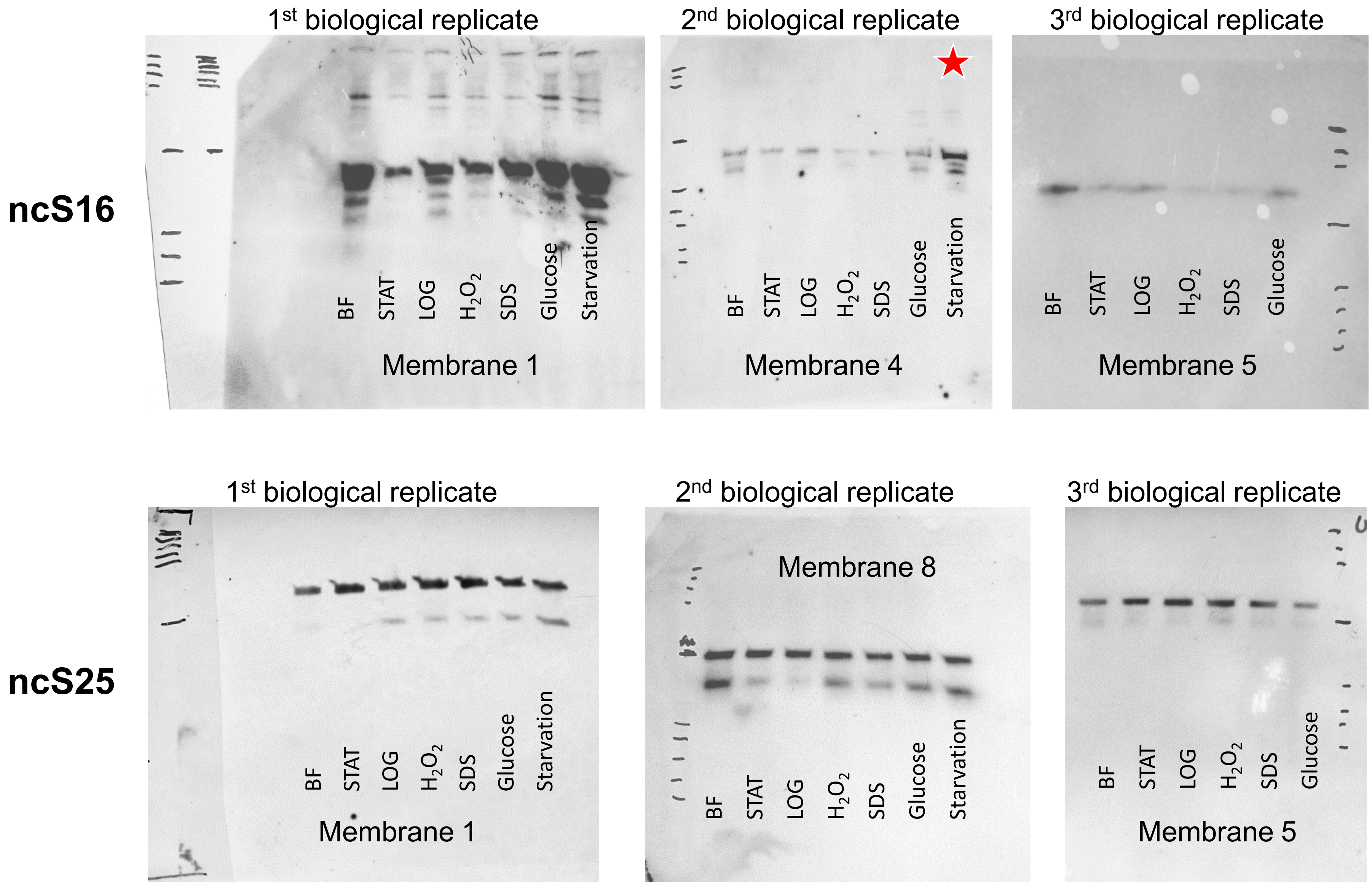

Figure S6, part 4

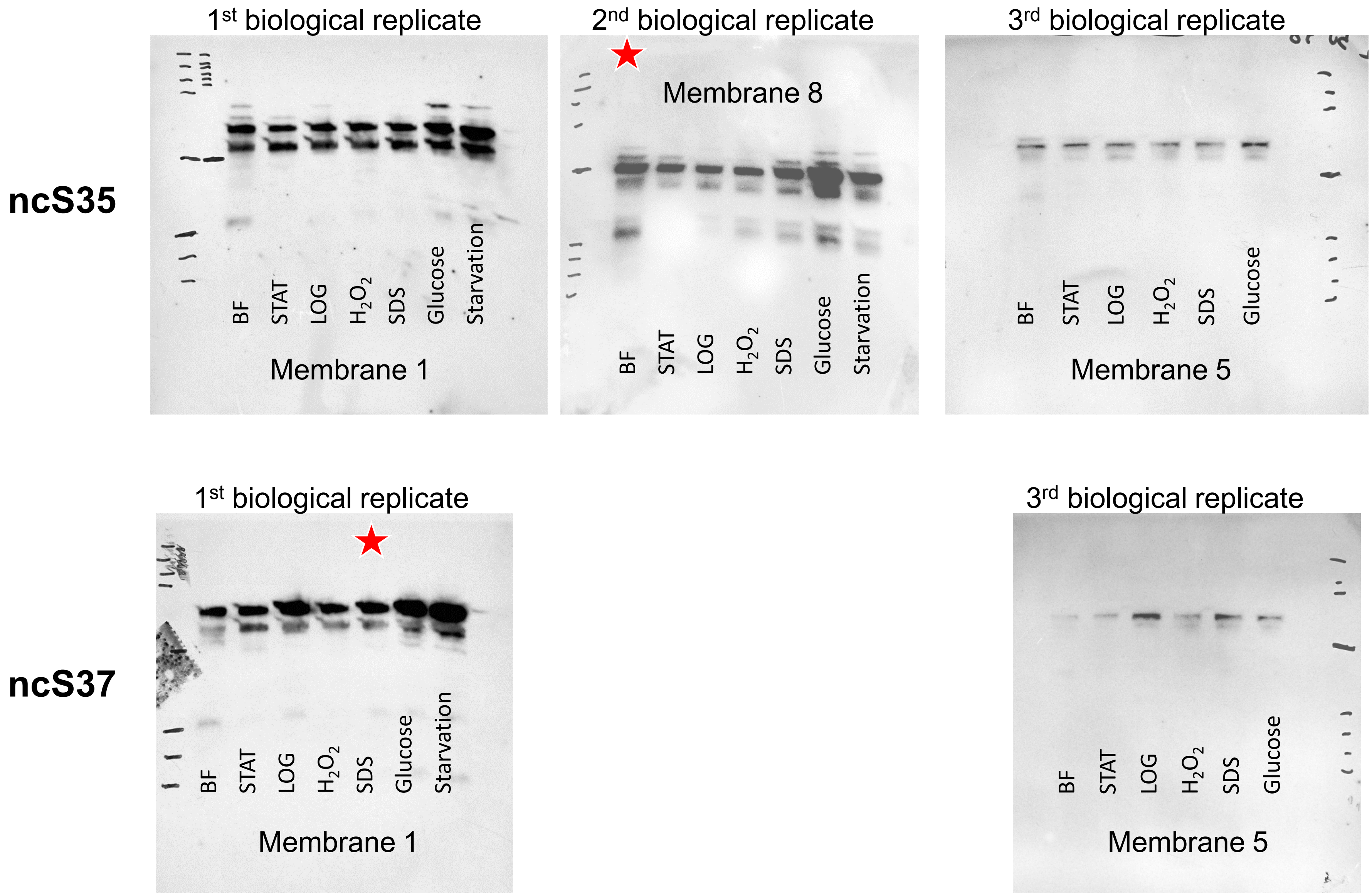

Figure S6, part 5

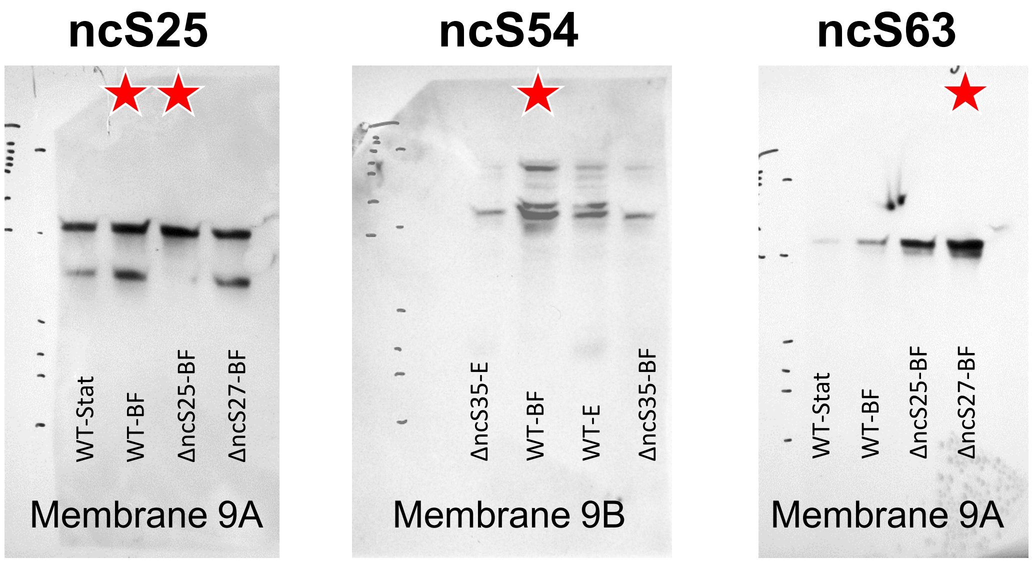

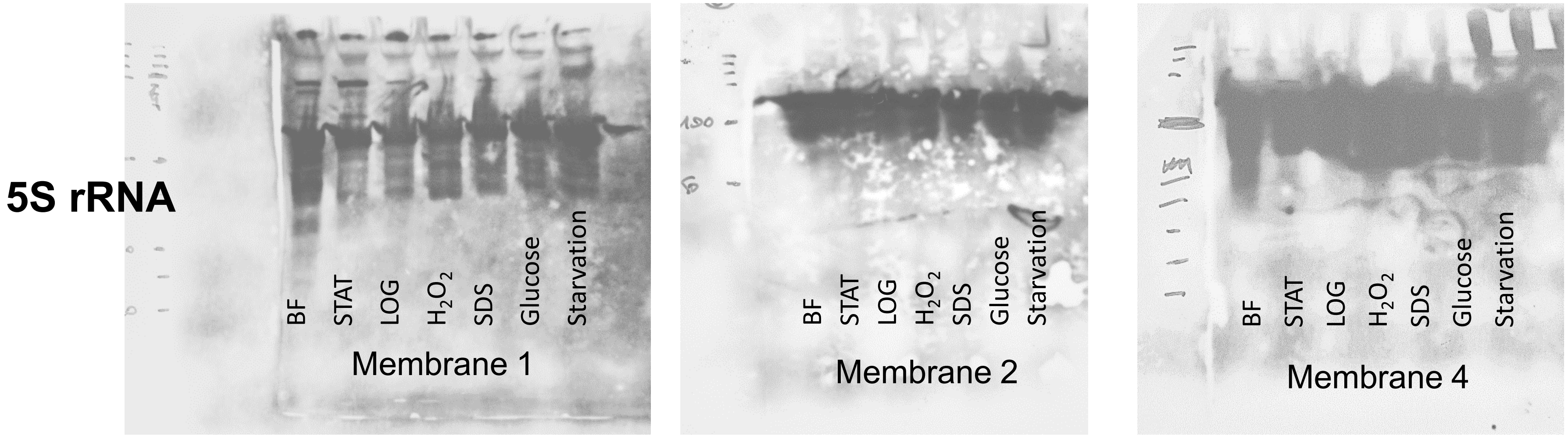

Figure S6, part 6

5S rRNA

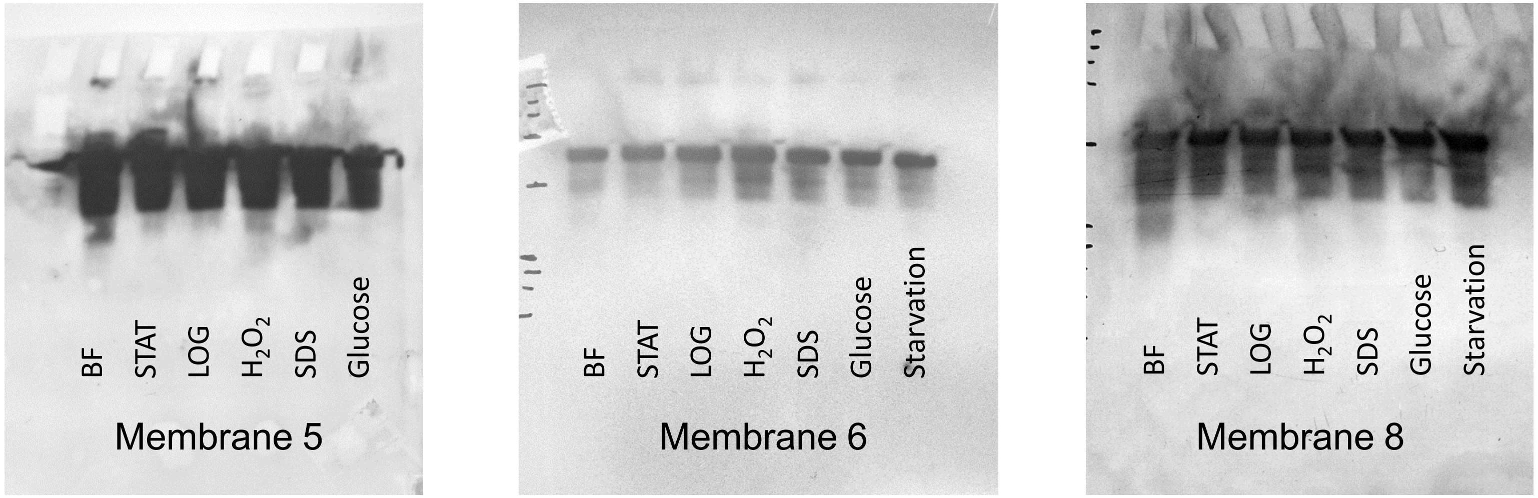

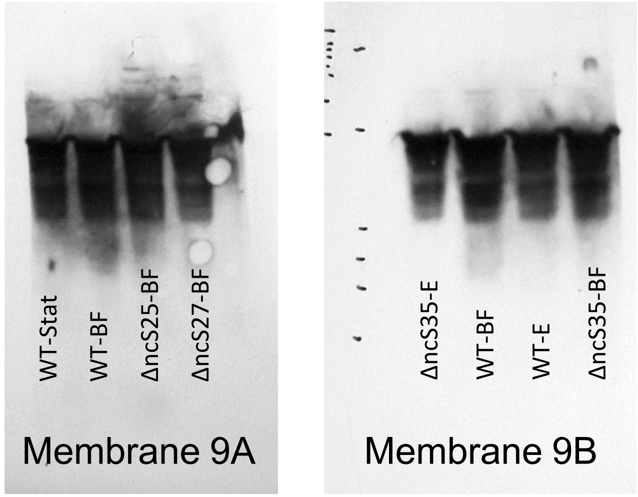

Figure S6: Full size images of Northern blots. RNA ladders were run alongside: 0.1-2 kb RNA ladder (Invitrogen) and a small RNA marker (Abnova, fragment sizes 20 to 100 bases). Lanes containing ladders were cut off the gel before blotting, stained with GelRed and bands documented to later transfer the position onto the developed x-ray films. X-ray films were then scanned to .jpg image on a Canon c5030i copy machine. Brightness was auto-adjusted for the whole image and images were converted to greyscale using irfanview software.
Lanes used for Figure 4 of the main manuscript are marked with a red star.
Abbreviations: BF: biofilm, STAT: stationary phase, LOG: exponential phase control condition, H_2O_2: cultures exposed to hydrogen peroxide, SDS: cultures exposed to sodium dodecyl sulfate, WT: *B. cenocepa*cia J2315 wild type, Δ: deletion mutant.

Doc. S1. Alignments of RACE results.

ncS01 3'RACE

```
>L13-A1
GCGAAACGAATTTGCAGGTGGAGAACGAGGAGACAATCGAGGCACAAAGTGCCCGGACCCCAAAGCGCTGTTGGACGGAATCCAACAGCGCTTTTTCATT
>L13-A2
GCGAAACGAATTTGCAGGTGGAGAACGAGGAGACAATCGAGGCACAAAGTGCCCGGACCCCAAAGCGCTGTTGGACGGAATCCAACAGCGCTTTTTCA
>L13-A4
GCGAAACGAATTTGCAGGTGGAGAACGAGGAGACAATCGAGGCACAAAGTGCCCGGACCCCAAAGCGCTGTTGGACGGAATCCAACAGCGCTTTTTCA
>L13-A5
CGCGAAACGAATTTGCAGGTGGAGAACGAGGAGACAATCGAGGCACAAAGTGCCCGGACCCCAAAGCGCTGTTGGACGGAATCCAACAGCGCTTTTTCA
>L13-A6
GCGAAACGAATTTGCAGGTGGAGAACGAGGAGACAATCGAGGCACAAAGTGCCCGGACCCCAAAGCGCTGTTGGACGGAATCCAACAGCGCTTTTTCA
>L13-A7
GCGAAACGAATTTGCAGGTGGAGAACGAGGAGACAATCGAGGCACAAAGTGCCCGGACCCCAAAGCGCTGTTGGACGGAATCCAACAGCGCTTTTTCA
>L13-A8
GCGAAACGAATTTGCAGGTGGAGAACGAGGAGACAATCGAGGCACAAAGTGCCCGGACCCCAAAGCGCTGTTGGACGGAATCCAACAGCGCTTTTTCA
```

```
L13-A5      CGCGAAACGAATTTGCAGGTGGAGAACGAGGAGACAATCGAGGCACAAAGTGCCCGGACC
L13-A1      -GCGAAACGAATTTGCAGGTGGAGAACGAGGAGACAATCGAGGCACAAAGTGCCCGGACC
L13-A2      -GCGAAACGAATTTGCAGGTGGAGAACGAGGAGACAATCGAGGCACAAAGTGCCCGGACC
L13-A4      -GCGAAACGAATTTGCAGGTGGAGAACGAGGAGACAATCGAGGCACAAAGTGCCCGGACC
L13-A6      -GCGAAACGAATTTGCAGGTGGAGAACGAGGAGACAATCGAGGCACAAAGTGCCCGGACC
L13-A7      -GCGAAACGAATTTGCAGGTGGAGAACGAGGAGACAATCGAGGCACAAAGTGCCCGGACC
L13-A8      -GCGAAACGAATTTGCAGGTGGAGAACGAGGAGACAATCGAGGCACAAAGTGCCCGGACC
             ***********************************************************

L13-A5      CCAAAGCGCTGTTGGACGGAATCCAACAGCGCTTTTTCA--
L13-A1      CCAAAGCGCTGTTGGACGGAATCCAACAGCGCTTTTTCATT
L13-A2      CCAAAGCGCTGTTGGACGGAATCCAACAGCGCTTTTTCA--
L13-A4      CCAAAGCGCTGTTGGACGGAATCCAACAGCGCTTTTTCA--
L13-A6      CCAAAGCGCTGTTGGACGGAATCCAACAGCGCTTTTTCA--
L13-A7      CCAAAGCGCTGTTGGACGGAATCCAACAGCGCTTTTTCA--
L13-A8      CCAAAGCGCTGTTGGACGGAATCCAACAGCGCTTTTTCA--
```

```
          ***************************************
```

ncS02 3'RACE

```
>K10-D1-M13_R.ab1
agccagtcggtcacgtaccgaagcccttccgggggggcatccctgttggagtcgtttccggcccccgccggactcggtttcatctttggtctcctcgcgctaaccc
cgtagcgtgtggtttttagcgggctaataagcccgcttttttttcg
>K10-D2-M13_R.ab1
tgccagtcggtcacgtaccgaagcccttccgggggggcatccctgttggagtcgtttccgacccccgccggactcggtttcatctttggtctcctcgcgctaaccc
catagcgtgtggtttttagcgggctaataagcccgctttttttcg
>K10-D3-M13_R.ab1
agccagtcggtcacgtaccgaagcccttccgggggggcatccctgttggagtcgtttccggcccccgccggactcggtttcatctttggtctcctcgcgctaaccc
cgtagcgtgtggtttttagcgggctaataagcccgcttttttttcg
>K10-D4-M13_R.ab1
agccagtcggtcacgtaccgaagcccttccgggggggcatccctgttggagtcgtttccggcccccgccggactcggtttcatctttggtctcctcgcgctaaccc
cgtagcgtgtggtttttagcgggctaataagcccgcttttttttcg
```

```
K10-D1-M13_R.AB1      AGCCAGTCGGTCACGTACCGAAGCCCTTCCGGGGGGGCATCCCTGTTGGAGTCGTTTCCGG
K10-D3-M13_R.AB1      AGCCAGTCGGTCACGTACCGAAGCCCTTCCGGGGGGGCATCCCTGTTGGAGTCGTTTCCGG
K10-D4-M13_R.AB1      AGCCAGTCGGTCACGTACCGAAGCCCTTCCGGGGGGGCATCCCTGTTGGAGTCGTTTCCGG
K10-D2-M13_R.AB1      TGCCAGTCGGTCACGTACCGAAGCCCTTCCGGGGGGGCATCCCTGTTGGAGTCGTTTCCGA
                       ***********************************************************

K10-D1-M13_R.AB1      CCCCCCGCCGGACTCGGTTTCATCTTTGGTCTCCTCGCGCTAACCCCGTAGCGTGTGGTT
K10-D3-M13_R.AB1      CCCCCCGCCGGACTCGGTTTCATCTTTGGTCTCCTCGCGCTAACCCCGTAGCGTGTGGTT
K10-D4-M13_R.AB1      CCCCCCGCCGGACTCGGTTTCATCTTTGGTCTCCTCGCGCTAACCCCGTAGCGTGTGGTT
K10-D2-M13_R.AB1      CCCCCCGCCGGACTCGGTTTCATCTTTGGTCTCCTCGCGCTAACCCCATAGCGTGTGGTT
                      *********************************************** ************

K10-D1-M13_R.AB1      TTTAGCGGGCTAATAAGCCCGCTTTTTTTCG
K10-D3-M13_R.AB1      TTTAGCGGGCTAATAAGCCCGCTTTTTTTCG
K10-D4-M13_R.AB1      TTTAGCGGGCTAATAAGCCCGCTTTTTTTCG
K10-D2-M13_R.AB1      TTTAGCGGGCTAATAAGCCCGCTTTTTT-CG
                      **************************** **
```

ncS04 3'RACE

```
>LC4-A1
AGCTCCCCGCTTCTCCTCCCTGAGCGGGTGCCTGTCG
>LC4-A2
AGCTCCCCGCTTCTCCTCCCTGAGCGGGTGCCTGTCGTTTTTCGATTAGGCTGTTTGACCCGCCGTTCAACGGCGGGTTTTT
```

```
>LC4-A3
AGCTCCCCGCTTCTCCTCCCTGAGCGGGTGCCTGTCGTTTTTCGATTAGGCTGTTTGACCCGCCGTTCAACGGCGGGTTTTTTT
>LC4-A4
AGCTCCCCGCTTCTCCTCCCTGAGCGGGTGCCTGTCGTTTTTCGATTAGGCTGTTTGACCCGCCGTT
>LC4-A6
AGCTCCCCGCTTCTCCTCCCTGAGCGGGTGCCTGTCGTTT
>LC4-A7
CCTCCCTGAGCGGGTGCCTGTCGTTTTTCGATTAGGCTGTTTGACCCGCCGTTCAACGGCGGGTTTTTTTA
>LC4-A8
AGCTCCCCGCTTCTCCTCCCTGAGCGGGTGCCTGTCGTTT
>LC4-A11
AGCTCCCCGCTTCTCCTCCCTGAGCGGGTGCCTGTCGTTTTTCGATTAGGCTGTTTGACCCGCCGTTCAACGGCGGGTTTTTTTA
>LC4-B1
AGCTCCCCGCTTCTCCTCCCTGAGCGGGTGCCTGTCGTTTTTCGATTAGGCTGTTTGACCCGCCGTTCAACGGCGGGTTTTTTTA
>LC4-B3
AGCTCCCCGCTTCTCCTCCCTGAGCGGGT
>LC4-B5
AGCTCCCCGCTTCTCCTCCCTGAGCGGGTGCCTGTCGTTTTTCGATTAGGCTGTTTGACCCGCCGTTCAACGGCGGGTTTTTTTA
```

```
LC4-A7       --------------CCTCCCTGAGCGGGTGCCTGTCGTTTTTCGATTAGGCTGTTTGACC
LC4-A1       AGCTCCCCGCTTCTCCTCCCTGAGCGGGTGCCTGTCG-----------------------
LC4-A2       AGCTCCCCGCTTCTCCTCCCTGAGCGGGTGCCTGTCGTTTTTCGATTAGGCTGTTTGACC
LC4-A3       AGCTCCCCGCTTCTCCTCCCTGAGCGGGTGCCTGTCGTTTTTCGATTAGGCTGTTTGACC
LC4-A4       AGCTCCCCGCTTCTCCTCCCTGAGCGGGTGCCTGTCGTTTTTCGATTAGGCTGTTTGACC
LC4-A6       AGCTCCCCGCTTCTCCTCCCTGAGCGGGTGCCTGTCGTTT--------------------
LC4-A8       AGCTCCCCGCTTCTCCTCCCTGAGCGGGTGCCTGTCGTTT--------------------
LC4-A11      AGCTCCCCGCTTCTCCTCCCTGAGCGGGTGCCTGTCGTTTTTCGATTAGGCTGTTTGACC
LC4-B1       AGCTCCCCGCTTCTCCTCCCTGAGCGGGTGCCTGTCGTTTTTCGATTAGGCTGTTTGACC
LC4-B3       AGCTCCCCGCTTCTCCTCCCTGAGCGGGT-------------------------------
LC4-B5       AGCTCCCCGCTTCTCCTCCCTGAGCGGGTGCCTGTCGTTTTTCGATTAGGCTGTTTGACC
                           ***************

LC4-A7       CGCCGTTCAACGGCGGGTTTTTTTA
LC4-A1       -------------------------
LC4-A2       CGCCGTTCAACGGCGGGTTTTT---
LC4-A3       CGCCGTTCAACGGCGGGTTTTTTT-
LC4-A4       CGCCGTT------------------
LC4-A6       -------------------------
LC4-A8       -------------------------
LC4-A11      CGCCGTTCAACGGCGGGTTTTTTTA
```

```
LC4-B1        CGCCGTTCAACGGCGGGTTTTTTTA
LC4-B3        -------------------------
LC4-B5        CGCCGTTCAACGGCGGGTTTTTTTA
```

ncS06 3'RACE

```
>LC5-E1
GAGAGTCTGCATGCACGGCGGGGCCAATCGTCGTCGAACAACACATGCATGCAGGCCGACTTGCACATAAGCGACGTTCCGTCGTCTGACCGGGGAAGGCAGTGAT
CGGGCGGCGGCGGACGTTTTTTAA
>LC5-E2
GAGAGTCTGCATGCACGGCGGGGCCAATCGTCGTCGAACAACACATGCATGCAGGCCGACTTGCACATAAGCGACGTTCCGTCGTCTGACCGGGGAAGGCAGTGAT
CGGGCGGCGGCGGACGTTTTTT
>LC5-E3
GAGAGTCTGCATGCACGGCGGGGCCAATCGTCGTCGAACAACACATGCATGCAGGCCGACTTGCACATAAGCGACGTTCCGTCGTCTGACCGGGGAAGGCAGTGAT
CGGGCGGCGGCGGACGTT
>LC5-E4
GAGAGTCTGCATGCACGGCGGGGCCAATCGTCGTCGAACAACACATGCATGCAGGCCGACTTGCACATAAGCGACGTTCCGTCGTCTGACCGGGGAAGGCAGTGAT
CGGGCGGCGGCGGACGTTTTTTAATCCGGAATGTTTTTCCGGTCACCGTCGAAAGACGGTTTCGGGGGGTGAAT
>LC5-E5
GAGAGTCTGCATGCACGGCGGGGCCAATCGTCGTCGAACAACACATGCATGCAGGCCGACTTGCACATAAGCGACGTTCCGTCGTCTGACCGGGGAAGGCAGTGAT
CGGGCGGCGGCGGACGTTTTTTAA
>LC5-E6
GAGAGTCTGCATGCACGGCGGGGCCAATCGTCGTCGAACAACACATGCATGCAGGCCGACTTGCACATAAGCGACGTTCCGTCGTCTGACCGGGGAAGGCAGTGAT
CGGGCGGCGGCGGACGTTTTTTAA
>LC5-E7
GAGAGTCTGCATGCACGGCGGGGCCAATCGTCGTCGAACAACACATGCATGCAGGCCGACTTGCACATAAGCGACGTTCCGTCGTCTGACCGGGGAAGGCAGTGAT
CGGGCGGCGGCGGACGTTTTTTAATCCGGAATGTTTTTCCGGTCACCGTCGAAAGACGGTTTCGGGGGGTGA
>LC5-G2
GAGAGTCTGCATGCACGGCGGGGCCAATCGTCGTCGAACAACACATGCATGCAGGCCGACTTGCACATAAGCGACGTTCCGTCGTCTGACCGGGGAAGGCAGTGAT
CGGGCGGCGGCGGACGTTTTTTAA
>LC5-G3
GAGAGTCTGCATGCACGGCGGGGCCAATCGTCGTCGAACAACACATGCATGCAGGCCGACTTGCACATAAGCGACGTTCCGTCGTCTGACCGGGGAAGGCAGTGAT
CGGGCGGCGGCGGACGTTTTTT
>LC5-G5
GAGAGTCTGCATGCACGGCGGGGCCAATCGTCGTCGAACAACACATGCATGCAGGCCGACTTGCACATAAGCGACGTTCCGTCGTCTGACCGGGGAAGGCAGTGAT
CGGGCGGCGGCGGACGTTTTTTAA
```

>LC5-H1
GAGAGTCTGCATGCACGGCGGGGCCAATCGTCGTCGAACAACACATGCATGCAGGCCGACTTGCACATAAGCGACGTTCCGTCGTCTGACCGGGGAAGGCAGTGAT
CGGGCGGCGGCGGACGTTTTTTAA

```
LC5-E7      GAGAGTCTGCATGCACGGCGGGGCCAATCGTCGTCGAACAACACATGCATGCAGGCCGAC
LC5-E4      GAGAGTCTGCATGCACGGCGGGGCCAATCGTCGTCGAACAACACATGCATGCAGGCCGAC
LC5-H1      GAGAGTCTGCATGCACGGCGGGGCCAATCGTCGTCGAACAACACATGCATGCAGGCCGAC
LC5-G5      GAGAGTCTGCATGCACGGCGGGGCCAATCGTCGTCGAACAACACATGCATGCAGGCCGAC
LC5-G2      GAGAGTCTGCATGCACGGCGGGGCCAATCGTCGTCGAACAACACATGCATGCAGGCCGAC
LC5-E6      GAGAGTCTGCATGCACGGCGGGGCCAATCGTCGTCGAACAACACATGCATGCAGGCCGAC
LC5-E5      GAGAGTCTGCATGCACGGCGGGGCCAATCGTCGTCGAACAACACATGCATGCAGGCCGAC
LC5-E1      GAGAGTCTGCATGCACGGCGGGGCCAATCGTCGTCGAACAACACATGCATGCAGGCCGAC
LC5-E2      GAGAGTCTGCATGCACGGCGGGGCCAATCGTCGTCGAACAACACATGCATGCAGGCCGAC
LC5-E3      GAGAGTCTGCATGCACGGCGGGGCCAATCGTCGTCGAACAACACATGCATGCAGGCCGAC
LC5-G3      GAGAGTCTGCATGCACGGCGGGGCCAATCGTCGTCGAACAACACATGCATGCAGGCCGAC
            ************************************************************

LC5-E7      TTGCACATAAGCGACGTTCCGTCGTCTGACCGGGGAAGGCAGTGATCGGGCGGCGGCGGA
LC5-E4      TTGCACATAAGCGACGTTCCGTCGTCTGACCGGGGAAGGCAGTGATCGGGCGGCGGCGGA
LC5-H1      TTGCACATAAGCGACGTTCCGTCGTCTGACCGGGGAAGGCAGTGATCGGGCGGCGGCGGA
LC5-G5      TTGCACATAAGCGACGTTCCGTCGTCTGACCGGGGAAGGCAGTGATCGGGCGGCGGCGGA
LC5-G2      TTGCACATAAGCGACGTTCCGTCGTCTGACCGGGGAAGGCAGTGATCGGGCGGCGGCGGA
LC5-E6      TTGCACATAAGCGACGTTCCGTCGTCTGACCGGGGAAGGCAGTGATCGGGCGGCGGCGGA
LC5-E5      TTGCACATAAGCGACGTTCCGTCGTCTGACCGGGGAAGGCAGTGATCGGGCGGCGGCGGA
LC5-E1      TTGCACATAAGCGACGTTCCGTCGTCTGACCGGGGAAGGCAGTGATCGGGCGGCGGCGGA
LC5-E2      TTGCACATAAGCGACGTTCCGTCGTCTGACCGGGGAAGGCAGTGATCGGGCGGCGGCGGA
LC5-E3      TTGCACATAAGCGACGTTCCGTCGTCTGACCGGGGAAGGCAGTGATCGGGCGGCGGCGGA
LC5-G3      TTGCACATAAGCGACGTTCCGTCGTCTGACCGGGGAAGGCAGTGATCGGGCGGCGGCGGA
            ************************************************************

LC5-E7      CGTTTTTTAATCCGGAATGTTTTTCCGGTCACCGTCGAAAGACGGTTTCGGGGGGTGA--
LC5-E4      CGTTTTTTAATCCGGAATGTTTTTCCGGTCACCGTCGAAAGACGGTTTCGGGGGGTGAAT
LC5-H1      CGTTTTTTAA--------------------------------------------------
LC5-G5      CGTTTTTTAA--------------------------------------------------
LC5-G2      CGTTTTTTAA--------------------------------------------------
LC5-E6      CGTTTTTTAA--------------------------------------------------
LC5-E5      CGTTTTTTAA--------------------------------------------------
LC5-E1      CGTTTTTTAA--------------------------------------------------
LC5-E2      CGTTTTTT----------------------------------------------------
LC5-E3      CGTT--------------------------------------------------------
LC5-G3      CGTTTTTT----------------------------------------------------
            ****
```

ncS11 3'RACE

```
>LC6-A1
AACCGATACCGCCAAAGAGCCCGCCGCCCGAAGGCGCCCGCGTGCGCATGAAAAAAGGCCTTCATCTGGCGATGAAGGCCTTTTAC
>LC6-A3
AACCGATACCGCCAAAGAGCCCGCCGCCCGAAGGCGCCCGCGTGCGCATGAAAAAAGGCCTTCATCTGGCGATGAAGGCCTTTTACT
>LC6-A4
AACCGATACCGCCAAAGAGCCCGCCGCCCGAAGGCGCCCGCGTGCGCATGAAAAAAGGCCTTCATCTGGCGATGAAGGCCTTTTACT
>LC6-A7
GCAACCGATACCGCCAAAGAGCCCGCCGCCCGAAGGCGCCCGCGTGCGCATGAAAAAAGGCCTTCATCTGGCGATGAAGGCCTTTTACT
```

```
LC6-A1      --AACCGATACCGCCAAAGAGCCCGCCGCCCGAAGGCGCCCGCGTGCGCATGAAAAAAGG
LC6-A3      --AACCGATACCGCCAAAGAGCCCGCCGCCCGAAGGCGCCCGCGTGCGCATGAAAAAAGG
LC6-A4      --AACCGATACCGCCAAAGAGCCCGCCGCCCGAAGGCGCCCGCGTGCGCATGAAAAAAGG
LC6-A7      GCAACCGATACCGCCAAAGAGCCCGCCGCCCGAAGGCGCCCGCGTGCGCATGAAAAAAGG
              **********************************************************

LC6-A1      CCTTCATCTGGCGATGAAGGCCTTTTAC-
LC6-A3      CCTTCATCTGGCGATGAAGGCCTTTTACT
LC6-A4      CCTTCATCTGGCGATGAAGGCCTTTTACT
LC6-A7      CCTTCATCTGGCGATGAAGGCCTTTTACT
            ****************************
L1-A6      ----------------
L1-A7      ----------------
```

ncS16 3' RACE

```
>LC7-E1
GTCACCATTCTCCTCTTGTCGTTGTTGCTCCGGGGCCGTATCCGCCCCGGTTTTTTT
>LC7-E2
GTCACCATTCTCCTCTTGTCGTTGTTGCTCCGGGGCCGTATCCGCCCCGGTTTTTTT
>LC7-E3
GTCACCATTCTCCTCTTGTCGTTGTTGCTCCGGGGCCGTATCCGCCCCGGTTTTTT
>LC7-E4
GTCACCATTCTCCTCTTGTCGTTGTTGCTCCGGGGCCGTATCCGCCCCGG
```

```
LC7-E1      GTCACCATTCTCCTCTTGTCGTTGTTGCTCCGGGGCCGTATCCGCCCCGGTTTTTTT
LC7-E2      GTCACCATTCTCCTCTTGTCGTTGTTGCTCCGGGGCCGTATCCGCCCCGGTTTTTTT
LC7-E3      GTCACCATTCTCCTCTTGTCGTTGTTGCTCCGGGGCCGTATCCGCCCCGGTTTTTT-
```

```
LC7-E4       GTCACCATTCTCCTCTTGTCGTTGTTGCTCCGGGGCCGTATCCGCCCCGG-------
             **************************************************
```

S18 3' RACE

```
>L4-A2
CCATCGAAGGGCCTTGTCTGTAAGGGCTACGGCGTTCTGAGTGCAGCCATGGATCACGTGGCGGTGCGATGGAACCTTCACCGGCGATCGAACCCCGAATTTAGCG
TATTAGGAG
>L4-A7
CCATCGAAGGGCCTTGTCTGTAAGGGCTACGGCGTTCTGAGTGCA
>L4-A1
CATCGAAGGGCCTTGTCTGTAAGGGCTACGGCGTTCTGAGTGCAGCCATGGATCACGTGGCGGTGCGATGGAACCTTCACCGGCGATCGAACC
>L4-A6
CCATCGAAGGGCCTTGTCTGTAAGGGCTACGGCGTTCTGAGTGCAGCCATGGATCACGTGGCGGTGCGATGGAACCTTCACCGGCGATCGA
>L4-A8
CCATCGAAGGGCCTTGTCTGTAAGGGCTACGGCGTTCTGAGTGCAGCCATGGATCACGTGGCGGTGCGATGGAACCTTCACCGGCGATCGA
```

```
L4-A1      -CATCGAAGGGCCTTGTCTGTAAGGGCTACGGCGTTCTGAGTGCAGCCATGGATCACGTG
L4-A2      CCATCGAAGGGCCTTGTCTGTAAGGGCTACGGCGTTCTGAGTGCAGCCATGGATCACGTG
L4-A7      CCATCGAAGGGCCTTGTCTGTAAGGGCTACGGCGTTCTGAGTGCA---------------
L4-A6      CCATCGAAGGGCCTTGTCTGTAAGGGCTACGGCGTTCTGAGTGCAGCCATGGATCACGTG
L4-A8      CCATCGAAGGGCCTTGTCTGTAAGGGCTACGGCGTTCTGAGTGCAGCCATGGATCACGTG
            ********************************************

L4-A1      GCGGTGCGATGGAACCTTCACCGGCGATCGAACC---------------------
L4-A2      GCGGTGCGATGGAACCTTCACCGGCGATCGAACCCCGAATTTAGCGTATTAGGAG
L4-A7      -------------------------------------------------------
L4-A6      GCGGTGCGATGGAACCTTCACCGGCGATCGA------------------------
L4-A8      GCGGTGCGATGGAACCTTCACCGGCGATCGA------------------------
```

S18 5'RACE

```
>18-1
ATTTAGCGTATTAGGAGTGCTCTCATGGCACGCGTATGCCAAGTAACTGGGAAAGCGCCGATGAGCGGCAACAACGTTTCCCACGCCAACAACAAGACGAAGCGT
>18-2
ATTTAGCGTATTAGGAGTGCTCTCATGGCACGCGTATGCCAAGTAACTGGGAAAGCGCCGATGAGCGGCAACAACGTTTCCCACGCCAACAACAAGACGAAGCGT
>18-3
```

ATTTAGCGTATTAGGAGTGCTCTCATGGCACGCGTATGCCAAGTAACTGGGAAAGCGCCGATGAGCGGCAACAACGTTTCCCACGCCAACAACAAGACGAAGCGT
>18-4
CTGTCTTTTTTCCAACCAGTTCTAGCCATCGAAGGGCCTTGTCTGTAAGGGCTACGGCGTTCTGAGTGCAGCCATGGATCACGTGGCGGTGCGATGGAACCTTCAC
CGGCGATCGAACCCCGAATTTAGCGTATTAGGAGTGCTCTCATGGCACGCGTATGCCAAGTAACTGGGAAAGCGCCGATGAGCGGCAACAACGTTTCCCACGCCAA
CAACAAGACGAAGCGT
>18-7
CTGTCTTTTTTCCAACCAGTTCTAGCCATCGAAGGGCCTTGTCTGTAAGGGCTACGGCGTTCTGAGTGCAGCCATGGATCACGTGGCGGTGCGATGGAACCTTCAC
CGGCGATCGAACCCCGAATTTAGCGTATTAGGAGTGCTCTCATGGCACGCGTATGCCAAGTAACTGGGAAAGCGCCGATGAGCGGCAACAACGTTTCCCACGCCAA
CAACAAGACGAAGCGT
>18-6
GTTCTAGCCATCGAAGGGCCTTGTCTGTAAGGGCTACGGCGTTCTGAGTGCAGCCATGGATCACGTGGCGGTGCGATGGAACCTTCACCGGCGATCGAACCCCGAA
TTTAGCGTATTAGGAGTGCTCTCATGGCACGCGTATGCCAAGTAACTGGGAAAGCGCCGATGAGCGGCAACAACGTTTCCCACGCCAACAACAAGACGAAGCGT

```
18-1       ------------------------------------------------------------
18-2       ------------------------------------------------------------
18-3       ------------------------------------------------------------
18-4       CTGTCTTTTTTCCAACCAGTTCTAGCCATCGAAGGGCCTTGTCTGTAAGGGCTACGGCGT
18-7       CTGTCTTTTTTCCAACCAGTTCTAGCCATCGAAGGGCCTTGTCTGTAAGGGCTACGGCGT
18-6       ------------------GTTCTAGCCATCGAAGGGCCTTGTCTGTAAGGGCTACGGCGT


18-1       ------------------------------------------------------------
18-2       ------------------------------------------------------------
18-3       ------------------------------------------------------------
18-4       TCTGAGTGCAGCCATGGATCACGTGGCGGTGCGATGGAACCTTCACCGGCGATCGAACCC
18-7       TCTGAGTGCAGCCATGGATCACGTGGCGGTGCGATGGAACCTTCACCGGCGATCGAACCC
18-6       TCTGAGTGCAGCCATGGATCACGTGGCGGTGCGATGGAACCTTCACCGGCGATCGAACCC


18-1       ---ATTTAGCGTATTAGGAGTGCTCTCATGGCACGCGTATGCCAAGTAACTGGGAAAGCG
18-2       ---ATTTAGCGTATTAGGAGTGCTCTCATGGCACGCGTATGCCAAGTAACTGGGAAAGCG
18-3       ---ATTTAGCGTATTAGGAGTGCTCTCATGGCACGCGTATGCCAAGTAACTGGGAAAGCG
18-4       CGAATTTAGCGTATTAGGAGTGCTCTCATGGCACGCGTATGCCAAGTAACTGGGAAAGCG
18-7       CGAATTTAGCGTATTAGGAGTGCTCTCATGGCACGCGTATGCCAAGTAACTGGGAAAGCG
18-6       CGAATTTAGCGTATTAGGAGTGCTCTCATGGCACGCGTATGCCAAGTAACTGGGAAAGCG
              *********************************************************

18-1       CCGATGAGCGGCAACAACGTTTCCCACGCCAACAACAAGACGAAGCGT
18-2       CCGATGAGCGGCAACAACGTTTCCCACGCCAACAACAAGACGAAGCGT
18-3       CCGATGAGCGGCAACAACGTTTCCCACGCCAACAACAAGACGAAGCGT
18-4       CCGATGAGCGGCAACAACGTTTCCCACGCCAACAACAAGACGAAGCGT
```

```
18-7      CCGATGAGCGGCAACAACGTTTCCCACGCCAACAACAAGACGAAGCGT
18-6      CCGATGAGCGGCAACAACGTTTCCCACGCCAACAACAAGACGAAGCGT
          ************************************************
```

S27 3’ RACE

```
>L14-E2
GCGGCAACCTCCGAATGTCTCCTCCACCCTCCTCCTAAGGTGGATTAAGCCCGAACCAGCCGTTCGGGCTTTTTTTC
>L14-E3
GCGGCAACCTCCGAATGTCTCCTCCACCCTCCTCCTAAGGTGGATTAAGCCCGAACCAGCCGTTC
>L14-E4
GCGGCAACCTCCGAATGTCTCCTCCACCCTCCTCCTAAGGTGGATTAAGCCCGAACCAGCCGTTCGGGCTTTTTTTC
>L14-E10
GCGGCAACCTCCGAATGTCTCCTCCACCCTCCTCCTAAGGTGGATTAAGCCCGAACCAGCCGTTCGGGCTTTTTTTC
>L14-E11
GCGGCAACCTCCGAATGTCTCCTCCACCCTCCTCCTAAGGTGGATTAAGCCCGAACCAGCCGTTCGGGCTTTTTTTC
>L14-E12
GCGGCAACCTCCGAATGTCTCCTCCACCCTCCTCCTAAGGTGGATTAAGCCCGAACCAGCCGTTCGGGCTTTTTTTC
```

```
L14-E2        GCGGCAACCTCCGAATGTCTCCTCCACCCTCCTCCTAAGGTGGATTAAGCCCGAACCAGC
L14-E3        GCGGCAACCTCCGAATGTCTCCTCCACCCTCCTCCTAAGGTGGATTAAGCCCGAACCAGC
L14-E4        GCGGCAACCTCCGAATGTCTCCTCCACCCTCCTCCTAAGGTGGATTAAGCCCGAACCAGC
L14-E10       GCGGCAACCTCCGAATGTCTCCTCCACCCTCCTCCTAAGGTGGATTAAGCCCGAACCAGC
L14-E11       GCGGCAACCTCCGAATGTCTCCTCCACCCTCCTCCTAAGGTGGATTAAGCCCGAACCAGC
L14-E12       GCGGCAACCTCCGAATGTCTCCTCCACCCTCCTCCTAAGGTGGATTAAGCCCGAACCAGC
              ************************************************************

L14-E2        CGTTCGGGCTTTTTTTC
L14-E3        CGTTC------------
L14-E4        CGTTCGGGCTTTTTTTC
L14-E10       CGTTCGGGCTTTTTTTC
L14-E11       CGTTCGGGCTTTTTTTC
L14-E12       CGTTCGGGCTTTTTTTC
              *****
```

S33 3’ RACE

```
>K11-H5-M13_R.ab1
ATTCCACTTCGCCATTCGATAAAGAGTCGCGGCGCGGCATGCACGTACATGCCGCCTGCCGCGACCGTC
```

>K11-H7-M13_R.ab1
TCGCCATTCGATAAAGAGTCGCGGCGCGGCATGCACGTACATGCCGCCTGCCGCGACCGTCACCTCACCACACCACGACCGGGCAGCGCACCGACCGCGCCACGCA
T
>K11-A3-M13-FP.ab1
CGGCGCGGCATGCACGTACATGCCGCCTGCCGCGACCGTCACCTCACCACACCACGACCGGGCAGCGCACCGACCGCGCCACGCAT
>K11-H2-M13-FP.ab1
CATTCGATAAAGAGTCGCGGCGCGGCATGCACGTACATGCCGCCTGCCGCGACCGTCACCTCACCACACCACGACCGGGCAGCGCACCGACCGCGCCACGCAT
>K11-H3-M13-FP.ab1
TCGCCATTCGATAAAGAGTCGCGGCGCGGCATGCACGTACATGCCGCCTGCCGCGACCGTC
>K11-H4-M13-FP.ab1
CATTCGATAAAGAGTCGCGGCGCGGCATGCACGTACATGCCGCCTGCCGCGACCGTCACCTCACCACACCACGACCGGGCAGCGCACCGACCGCGCCACGCAT
>K11-H6-M13-FP.ab1
ATTCCACTTCGCCATTCGATAAAGAGTCGCGGCGCGGCATGCACGTACATGCCGCCTGCCGCGACCGTCACCTCACCACACCACGACCGGGCAGCGCACCGACCGC
GCCACGCAT

```
K11-H5-M13_R.ab1       ATTCCACTTCGCCATTCGATAAAGAGTCGCGGCGCGGCATGCACGTACATGCCGCCTGCC
K11-H3-M13-FP.ab1      --------TCGCCATTCGATAAAGAGTCGCGGCGCGGCATGCACGTACATGCCGCCTGCC
K11-H6-M13-FP.ab1      ATTCCACTTCGCCATTCGATAAAGAGTCGCGGCGCGGCATGCACGTACATGCCGCCTGCC
K11-H7-M13_R.ab1       --------TCGCCATTCGATAAAGAGTCGCGGCGCGGCATGCACGTACATGCCGCCTGCC
K11-A3-M13-FP.ab1      -----------------------------CGGCGCGGCATGCACGTACATGCCGCCTGCC
K11-H2-M13-FP.ab1      ------------CATTCGATAAAGAGTCGCGGCGCGGCATGCACGTACATGCCGCCTGCC
K11-H4-M13-FP.ab1      ------------CATTCGATAAAGAGTCGCGGCGCGGCATGCACGTACATGCCGCCTGCC
                                                    *******************************

K11-H5-M13_R.ab1       GCGACCGTC---------------------------------------------------
K11-H3-M13-FP.ab1      GCGACCGTC---------------------------------------------------
K11-H6-M13-FP.ab1      GCGACCGTCACCTCACCACACCACGACCGGGCAGCGCACCGACCGCGCCACGCAT
K11-H7-M13_R.ab1       GCGACCGTCACCTCACCACACCACGACCGGGCAGCGCACCGACCGCGCCACGCAT
K11-A3-M13-FP.ab1      GCGACCGTCACCTCACCACACCACGACCGGGCAGCGCACCGACCGCGCCACGCAT
K11-H2-M13-FP.ab1      GCGACCGTCACCTCACCACACCACGACCGGGCAGCGCACCGACCGCGCCACGCAT
K11-H4-M13-FP.ab1      GCGACCGTCACCTCACCACACCACGACCGGGCAGCGCACCGACCGCGCCACGCAT
                       *********
```

S33 5'RACE

>33-2
ATGCGACGGGGCCATCCAATAGCCATTCCACTTCGCCATTCGATAAAGAGTCGCGGCGCGGCATGCACGTACATGCCGCCTGCCGCGACCGTCACCTCACCACACC
ACGACCGGGCAGCGCACCGACCGCGCCACGCATGACGTCGAGCCGTACACGACGCATGCCGCGCCGCAGCAACATGCGCAGCATCCGAGCCGTCATTTGGCAAGCG
CGTTAACAACTTGTTTCGGAATGACCGGAACCGTGCCCGCCCGATGCGATCTCACTTGCACGCAGCACTGCTGCGCATCCCACTCAATCAATCACGACTCGAGGGA

GATAACGTGAACGTGAAATACTTACCGCTCATCGCATTGACCGTGGCAATTTCTGCTCATGCCGCTGAACCTGCCGTGCAAAACGTGGGACAAAGCCAGAAAGACG
C
>33-5
ACGCAGCACTGCTGCGCATCCCACTCAATCAATCACGACTCGAGGGAGATAACGTGAACGTGAAATACTTACCGCTCATCGCATTGACCGTGGCAATTTCTGCTCA
TGCCGCTGAACCTGCCGTGCAAAACGTGGGACAAAGCCAGAAAGACGC
>33-6
ACGCAGCACTGCTGCGCATCCCACTCAATCAATCACGACTCGAGGGAGATAACGTGAACGTGAAATACTTACCGCTCATCGCATTGACCGTGGCAATTTCTGCTCA
TGCCGCTGAACCTGCCGTGCAAAACGTGGGACAAAGCCAGAAAGACGC
>33-12
GCGCACCGACCGCGCCACGCATGACGTCGAGCCGTACACGACGCATGCCGCGCCGCAGCAACATGCGCAGCATCCGAGCCGTCATTTGGCAAGCGCGTTAACAACT
TGTTTCGGAATGACCGGAACCGTGCCCGCCCGATGCGATCTCACTTGCACGCAGCACTGCTGCGCATCCCACTCAATCAATCACGACTCGAGGGAGATAACGTGAA
CGTGAAATACTTACCGCTCATCGCATTGACCGTGGCAATTTCTGCTCATGCCGCTGAACCTGCCGTGCAAAACGTGGGACAAAGCCAGAAAGACGC
>33-13
ATGCGACGGGGCCATCCAATAGCCATTCCACTTCGCCATTCGATAAAGAGTCGCGGCGCGGCATGCACGTACATGCCGCCTGCCGCGACCGTCACCTCACCACACC
ACGACCGGGCAGCGCACCGACCGCGCCACGCATGACGTCGAGCCGTACACGACGCATGCCGCGCCGCAGCAACATGCGCAGCATCCGAGCCGTCATTTGGCAAGCG
CGTTAACAACTTGTTTCGGAATGACCGGAACCGTGCCCGCCCGATGCGATCTCACTTGCACGCAGCACTGCTGCGCATCCCACTCAATCAATCACGACTCGAGGGA
GATAACGTGAACGTGAAATACTTACCGCTCATCGCATTGACCGTGGCAATTTCTGCTCATGCCGCTGAACCTGCCGTGCAAAACGTGGGACAAAGCCAGAAAGACG
C
>S33-16
GCGACCGTCACCTCACCACACCACGACCGGGCAGCGCACCGACCGCGCCACGCATGACGTCGAGCCGTACACGACGCATGCCGCGCCGCAGCAACATGCGCAGCAT
CCGAGCCGTCATTTGGCAAGCGCGTTAACAACTTGTTTCGGAATGACCGGAACCGTGCCCGCCCGATGCGATCTCACTTGCACGCAGCACTGCTGCGCATCCCACT
CAATCAATCACGACTCGAGGGAGATAACGTGAACGTGAAATACTTACCGCTCATCGCATTGACCGTGGCAATTTCTGCTCATGCCGCTGAACCTGCCGTGCAAAAC
GTGGGACAAAGCCAGAAAGACGC

```
33-2        ATGCGACGGGGCCATCCAATAGCCATTCCACTTCGCCATTCGATAAAGAGTCGCGGCGCG
33-5        ------------------------------------------------------------
33-6        ------------------------------------------------------------
33-12       ------------------------------------------------------------
33-13       ATGCGACGGGGCCATCCAATAGCCATTCCACTTCGCCATTCGATAAAGAGTCGCGGCGCG
S33-16      ------------------------------------------------------------


33-2        GCATGCACGTACATGCCGCCTGCCGCGACCGTCACCTCACCACACCACGACCGGGCAGCG
33-5        ------------------------------------------------------------
33-6        ------------------------------------------------------------
33-12       ---------------------------------------------------------GCG
33-13       GCATGCACGTACATGCCGCCTGCCGCGACCGTCACCTCACCACACCACGACCGGGCAGCG
```

```
S33-16      ------------------------GCGACCGTCACCTCACCACACCACGACCGGGCAGCG

33-2        CACCGACCGCGCCACGCATGACGTCGAGCCGTACACGACGCATGCCGCGCCGCAGCAACA
33-5        ------------------------------------------------------------
33-6        ------------------------------------------------------------
33-12       CACCGACCGCGCCACGCATGACGTCGAGCCGTACACGACGCATGCCGCGCCGCAGCAACA
33-13       CACCGACCGCGCCACGCATGACGTCGAGCCGTACACGACGCATGCCGCGCCGCAGCAACA
S33-16      CACCGACCGCGCCACGCATGACGTCGAGCCGTACACGACGCATGCCGCGCCGCAGCAACA

33-2        TGCGCAGCATCCGAGCCGTCATTTGGCAAGCGCGTTAACAACTTGTTTCGGAATGACCGG
33-5        ------------------------------------------------------------
33-6        ------------------------------------------------------------
33-12       TGCGCAGCATCCGAGCCGTCATTTGGCAAGCGCGTTAACAACTTGTTTCGGAATGACCGG
33-13       TGCGCAGCATCCGAGCCGTCATTTGGCAAGCGCGTTAACAACTTGTTTCGGAATGACCGG
S33-16      TGCGCAGCATCCGAGCCGTCATTTGGCAAGCGCGTTAACAACTTGTTTCGGAATGACCGG

33-2        AACCGTGCCCGCCCGATGCGATCTCACTTGCACGCAGCACTGCTGCGCATCCCACTCAAT
33-5        -------------------------------ACGCAGCACTGCTGCGCATCCCACTCAAT
33-6        -------------------------------ACGCAGCACTGCTGCGCATCCCACTCAAT
33-12       AACCGTGCCCGCCCGATGCGATCTCACTTGCACGCAGCACTGCTGCGCATCCCACTCAAT
33-13       AACCGTGCCCGCCCGATGCGATCTCACTTGCACGCAGCACTGCTGCGCATCCCACTCAAT
S33-16      AACCGTGCCCGCCCGATGCGATCTCACTTGCACGCAGCACTGCTGCGCATCCCACTCAAT
                                           *****************************

33-2        CAATCACGACTCGAGGGAGATAACGTGAACGTGAAATACTTACCGCTCATCGCATTGACC
33-5        CAATCACGACTCGAGGGAGATAACGTGAACGTGAAATACTTACCGCTCATCGCATTGACC
33-6        CAATCACGACTCGAGGGAGATAACGTGAACGTGAAATACTTACCGCTCATCGCATTGACC
33-12       CAATCACGACTCGAGGGAGATAACGTGAACGTGAAATACTTACCGCTCATCGCATTGACC
33-13       CAATCACGACTCGAGGGAGATAACGTGAACGTGAAATACTTACCGCTCATCGCATTGACC
S33-16      CAATCACGACTCGAGGGAGATAACGTGAACGTGAAATACTTACCGCTCATCGCATTGACC
            ************************************************************

33-2        GTGGCAATTTCTGCTCATGCCGCTGAACCTGCCGTGCAAAACGTGGGACAAAGCCAGAAA
33-5        GTGGCAATTTCTGCTCATGCCGCTGAACCTGCCGTGCAAAACGTGGGACAAAGCCAGAAA
33-6        GTGGCAATTTCTGCTCATGCCGCTGAACCTGCCGTGCAAAACGTGGGACAAAGCCAGAAA
33-12       GTGGCAATTTCTGCTCATGCCGCTGAACCTGCCGTGCAAAACGTGGGACAAAGCCAGAAA
33-13       GTGGCAATTTCTGCTCATGCCGCTGAACCTGCCGTGCAAAACGTGGGACAAAGCCAGAAA
S33-16      GTGGCAATTTCTGCTCATGCCGCTGAACCTGCCGTGCAAAACGTGGGACAAAGCCAGAAA
            ************************************************************

33-2        GACGC
```

```
33-5         GACGC
33-6         GACGC
33-12        GACGC
33-13        GACGC
S33-16       GACGC
             *****
```

S35 3’ RACE

```
>L6-A3
GACAAGTGCGCGCAACGATTCCGCTTCCGACGCACATATGTCCATGGCACGCAGGAAGCGAATCCGGGACTCTCAACCCCCATCGGGAGACCCGGAGCCCTGAGCGAACATCCACG
CGGCTGTGCACCGGCCCCGTGTGGATGT
>L6-A5
GACAAGTGCGCGCAACGATTCCGCTTCCGACGCACATATGTCCATGGCACGCAGGAAGCGAATCCGGGACTCTCAACCCCCATCGGGAGACCCGGAGCCCTGAGCGAACATCCACG
CGGCTGTGCACCGGCCCCGTGT
>L6-A6
GACAAGTGCGCGCAACGATTCCGCTTCCGACGCACATATGTCCATGGCACGCAGGAAGCGAATCCGGGACTCTCAACCCCCATCGGGAGACCCGGAGCCCTGAGCGAACATCCACG
CGGCTGTGCACCGGCCCCGTGTGGATGTTCGCTGATTTTT
>L7-A1
ACATATGTCCATGGCACGCAGGAAGCGAATCCGGGACTCTCAACCCCCATCGGGAGACCCGGAGCCCTGAGCGAACATCCACGCGGCTGTGCACCGGCCCCGTGTGGATGTTCGCT
GATTTTT
>L7-A3
ACATATGTCCATGGCACGCAGGAAGCGAATCCGGGACTCTCAACCCCCATCGGGAGACCCGGAGCCCTGAGCGAACATCCACGCGGCTGTGCACCGGCCCCGTGTGGATGTTCGCT
GATTTTT
>L7-A4
ACATATGTCCATGGCACGCAGGAAGCGAATCCGGGACTCTCAACCCCCATCGGGAGACCCGGAGCCCTGAGCGAACATCCACGCGGCTGTGCACCGGCCCCGTGTGGATGTTCGCT
GATTTTT
```

```
L7-A4       ---------------------------------ACATATGTCCATGGCACGCAGGAAGCG
L7-A3       ---------------------------------ACATATGTCCATGGCACGCAGGAAGCG
L7-A1       ---------------------------------ACATATGTCCATGGCACGCAGGAAGCG
L6-A3       GACAAGTGCGCGCAACGATTCCGCTTCCGACGCACATATGTCCATGGCACGCAGGAAGCG
L6-A5       GACAAGTGCGCGCAACGATTCCGCTTCCGACGCACATATGTCCATGGCACGCAGGAAGCG
L6-A6       GACAAGTGCGCGCAACGATTCCGCTTCCGACGCACATATGTCCATGGCACGCAGGAAGCG
                                             ***************************

L7-A4       AATCCGGGACTCTCAACCCCCATCGGGAGACCCGGAGCCCTGAGCGAACATCCACGCGGC
L7-A3       AATCCGGGACTCTCAACCCCCATCGGGAGACCCGGAGCCCTGAGCGAACATCCACGCGGC
L7-A1       AATCCGGGACTCTCAACCCCCATCGGGAGACCCGGAGCCCTGAGCGAACATCCACGCGGC
L6-A3       AATCCGGGACTCTCAACCCCCATCGGGAGACCCGGAGCCCTGAGCGAACATCCACGCGGC
```

```
L6-A5      AATCCGGGACTCTCAACCCCCATCGGGAGACCCGGAGCCCTGAGCGAACATCCACGCGGC
L6-A6      AATCCGGGACTCTCAACCCCCATCGGGAGACCCGGAGCCCTGAGCGAACATCCACGCGGC
           ************************************************************

L7-A4      TGTGCACCGGCCCCGTGTGGATGTTCGCTGATTTTT
L7-A3      TGTGCACCGGCCCCGTGTGGATGTTCGCTGATTTTT
L7-A1      TGTGCACCGGCCCCGTGTGGATGTTCGCTGATTTTT
L6-A3      TGTGCACCGGCCCCGTGTGGATGT------------
L6-A5      TGTGCACCGGCCCCGTGT------------------
L6-A6      TGTGCACCGGCCCCGTGTGGATGTTCGCTGATTTTT
           ******************
```

Doc. S2. sRNA sequences, with probe and processing sites indicated, and with secondary structures in Vienna format.

>ncS01

AGUUCUGAUGCACCGCGAAACGA↓AUUUGCAGGUGGAGA↓ACGAGGAGACAAUCGAGGCACAAAGUGCCCGGACCCCAAAGCGCUGUUGGACGGAA
UCCAACAGCGCUUUUUC

.(((((....(((((((((.....))))).)))).))))).........(((.(((((...))))))))).....((((((((((((((.....)))
)))))))))))...

>ncS02

CGGAUGCGGUCUCGUGUGCCAAGUGUGAGCGGACGCGAAGCGCUGCGAGCCAGUCGGUCACGUACCGAAGCCCUUCCGGGGGGCAUCCCUG↓UUGG
AGUCGUUUCCGGCCCCCCGCCGGACUCGGUUUCAUCUUUGGUCUCCUCGCGCUAACCCCGUAGCGUGUGGUUUUUAGCGGGCUAAUAAGCCCGCUUU
UUUUCG

.((((((((.(((((((((...((((......))))...)))).)))))))..(((((.....)))))..(((.....))).))))))....((((.
.((..(((((.......)))))..))..))))..........((.(((((((......))))))).)).....((((((((....))))))))....
....

>ncS03

GUCUCCUCCAUGUCUCCUCUGAUAUGGAUUCAGCCCGCCACUUAGGCGGGCUUUUUUUU

......((((((((......))))))))...((((((((.....))))))))........

>ncS04

CGAGCGGCACGCGUUGCGUGCAGCUCCCCGCUUCUCCUCCCUGAGCGGGUGCCUGUCGUUUUUCGAUUAGGCUGUUUGACCCGCCGUUCAACGGCGG
GUUUUUUU
.((((.((((((...)))))).))))((((((.(........))))))).((((((((.....))).))))).....(((((((((.....))))))
))).....

>ncS05

GUCUCCUCCAUGUCUCCUCCUGAUAUGGAUUAAGCCCGUUCCGCACUGCGUGAACGGGCUUUUUUUC
......((((((((.......))))))))..((((((((((((...))).)))))))))......

>ncS06

AUCCCGUUCGGCCGAUAUCUAUUCUUCACC↓CCACAAGUUGAAAAAAUGAAAAGAAUAGAUAAG↓UUCUACGGGGUAGCAGA↓AAUAAAGCGCGG
GGUGAGCGUCGCCGGCACGUGAAACGCGUGCCGGCCGCGGAGAGUCUGCAUGCACGGCGGGGCCAAUCGUCGUCGAACAACACAUGCAUGCAGGCCG
ACUUGCACAUAAGCGACGUUCCGUCGUCUGACCGGGGAAGGCAGUGAUCGGGCGGCGGCGGACGUUUUUU
(((((((..(((...((((((((((((........................)))))))))))).)))..))))))).............(((((....
.(((.(((((((((((...))))))))))).)))....((((((((((((((((((....)).))))))).........)))))))))))...))))
)........(((((.((((((.(((.((.((...........)).))))))))))))))))....

>ncS11

CCCAUUUCGUGAAGGAAGUUCUGAGCGAAAUCAGGUGGCCGCAUAUAUUGCGAGCCAGAUAGGGCAGAACGCGCAGUCCGUGCAGACCACGGCAGAG
UCGCAUUUGCGUUGCUCGAGGCGCAACCGAUACCGCCAAAGAGCCCGCCGCCCGAAGGCGCCCGCGUGCGCAUGAAAAAAGGCCUUCAUCUGGCGAU
GAAGGCCUUUUACU
(((.((((((..(((....)))..)))))).....((((((((.....)))).))))....)))......((((.(.(((((.....)))))).....
.(((....(((..((((..((((...........))))...)))).)))((((....))))...))).)))).....((((((((((((((....)))
))))))))))...

>ncS16

GUACGCGGCUGGUGUUGCCGGCGUCACCA<u>UUCUCCUCUUGUCGUUGUU</u>GCUCCGGGGCCGUAUCCGCCCCGGUUUUUUUUU

(((((((.((((.....))))))))).)).......................(((((((.......))))))).........

>ncS25

UCUGCUUUCC<u>GAAGCAGACUUUUUCGUUGA</u>CGGAAAAGAUCUCCAAACCUUUGUCGGCGCGACUUCCCAGUCGCGCUUUUUUUUU

(((((((....)))))))((((((((....)))))))).................(((((((((....)))))))))........

>ncS27

AUUGGUUGUCGCGCUGCGGCAACCUCCGA↓AUGUCUCCUCCACCCUCCUCCUAAGGUGGAUUAAGCCCGAACCAGCCGUUCGGGCUUUUUUUC

...(((((((((...))))))))).............((((((..........))))))..((((((((((.....))))))))))......

>ncS54

ACGUUUUCGAAACAUCGUUUCAAGA<u>GUUUAACCGGCAACGAUGU</u>UUCGCGCAGU↓CUCUCAGGUUCUAGCACGGCCCUCGGAAUGGCUAGAACUUU

UUUAGCGCCACGGUCUCCGUGGCGCUUUUUUUUU

..((...((((((((((((((............)).)))))))))))).)).........((((((((((....((...))....))))))))))...

.((((((((((...)))))))))).......

>ncS63

GUAUUGUGGGGACCACCUCCCUGAGAGGUGUUGGGCAGUAGCCAGCGUAACGGCUUGCACGC<u>GAGAUCUAGACCCCUUCGUG</u>CAAACACACUCAAGC

AGCCGUUGCAGCAAGCCAAGCCACUUUUU

.......((((......)))).((((((((...((((....))).(((((((((((((((((.(((..........))))))))).............

))))))))).)).........))))))))

>ncS35

AUGGCGCGACGACAAGUGCGCGCAACGAU↓UCCGCUUCCGACGCACAUAUGUCCAUGGCACGCAGGAAGC<u>GAAUCCGGGACUCUCAA</u>CCCCCAUCG

GGAGACCCGGAGCCCUGAGCGAACAUCCACGCGGCUGUGCACCGGCCCCGUGUGGAUGUUCGCUGAUUUUUUU

...(((((((.....)).)))))........(((((((..((..(((......)))...))..)))))))..((((((.(((((..........)))

)).))))))......((((((((((((((((((((.....)))))..)))))))))))))))).........

>ncS37

GCCGGACCGCGCUUCCGGUACAUCGCCUCCCCGCCCGCGCAGGAUCCCUGCGGGCACCCUCCG↓UCCUCCGGCGAUGUAUCCCGUGACUGGGCGAG

CAGACACGGCGCGCGGGCCGAUUUUCCGCCCCGGACGUCCCCGUCCGGGGCUUUUUGU

..(((.((((((....(((((((((((.....((((..(((((...))))))))).(.....)......)))))))))))(((((.(((......))

).))))).)))))).))).......((((((((((....)))))))))).......

>ncS62

CUGUCUCCUCCAUGUCUCCAAACAUGGAUUCAGCCCGCUCCAGCAGCGGGCUUUUUU

........(((((((......)))))))...((((((((.....)))))))).....

underlined and yellow: probe alignment site, arrows, shaded blue: processing site

Doc. S3. sRNA-mRNA interaction diagrams.

Interactions of ncS03 with predicted target mRNAs

```
            -21                             8       BCAL3365
             |                              |
5'-CGU...CGAAC     UA          UGCC         CCGUC...CUG-3'
              AAUUC     GGAGGAG    ACAUGGGGG
              |||:|     :||||||    ||||||:||
              UUAGG     UCUCCUC    UGUACCUCC
3'-UUU...CCGAC     UAUAG                    UCUG-5'
             |                              |
             31                             4       ncS03
```

```
            -13     -5        BCAL0184
             |       |
5'-CCG...UUCGG       GAGUC...CGA-3'
              AGGAGAC
              |||||||
              UCCUCUG
3'-UUU...GUACC       -5'
             |      |
             8      1         ncS03
```

```
            -16                               10     BCAL0925
             |                               |
5'-CCU...AAGCC             U                 AUGCA...UCG-3'
              AAUCCA   CGGA GGAGAU   GA GAGAC
              ||||||   |:|| |||||:   || |||||
              UUAGGU   GUCU CCUCUG   CU CUCUG
3'-UUU...CCGAC      AUA           UAC  C     -5'
             |                              |
             31                             1       ncS03
```

```
            -12                     12      BCAM1274
             |                      |
5'-AGC...GACGC       GAAC     C     AAUCA...GCG-3'
              AGGAGAC    AUGGG GAGAC
              |||||||    ||||: |||||
              UCCUCUG    UACCU CUCUG
3'-UUU...UAGUC                C     -5'
             |                     |
             19                    1        ncS03
```

```
            -25                        -4         BCAM2500
             |                          |
5'-CCG...AGACA  GAA                     GACCA...UCG-3'
              CA    AGA GAGAC    AGGAGAC
              ||    ||| |||||    |||||||
              GU    UCU CUCUG    UCCUCUG
3'-UUU...CUUAG  AUAG   C     UACC       -5'
             |                         |
             27                        1          ncS03
```

```
            -15        -4              BCAL3041
             |          |
5'-GCU...CAGUC          AAGUA...GGU-3'
              UGGAGGAGAU
              |||||||||:
              ACCUCCUCUG
3'-UUU...UCUGU          -5'
             |         |
             11        1               ncS03
```

```
            -9                    14           BCAL3043
             |                     |
5'-UCG...CAGUC             AUC     CCACG...CGC-3'
              CGGAGGAGAUGUG   GAGAU
              |:|||||||::||   ||||:
              GUCUCCUCUGUAC   CUCUG
3'-UUU...GUAUA             CUC     -5'
             |                    |
             22                   1            ncS03
```

Interactions of ncS63 with predicted target mRNAs

```
            -67         -55        BCAL3299
             |           |
5'-ACG...UUACC           UCCAC...AUC-3'
              CCCCGCAAUGC
              ||||:||||:|
              GGGGUGUUAUG
3'-UUU...CACCA           -5'
             |          |
             12         1          ncS63
```

```
             62                         88       BCAM0969
             |                          |
5'-AAG...CGGGC    CC       GC           AACUG...CGC-3'
              GGCU  GGCUUGC  GCGGC GCUGC
              ||||  |||||||  ||::| |||||
              CCGA  CCGAACG  CGUUG CGACG
    3'-UUUUUCA    A        A      C     AACUC...AUG-5'
             |                          |
             120                        95       ncS63
```

```
            -37                            -12      BCAL2141
             |                              |
5'-CGA...UCUGC      U   UUU                 AGCGU...CGU-3'
              GUGUUU C C       GUCUA  UCUCGCG
              |||||| | |       |||||  |||||||
              CACAAA G G       CAGAU  AGAGCGC
3'-UUU...ACUCA      C U CUUCCC     CU       ACGUU...AUG-5'
             |                              |
             90                             59       ncS63
```

```
             1                  20       BCAM1216
             |                  |
5'-CGC...CGCCA           AA     AGAAC...CGC-3'
              UGCUCGACGCC  UCUCA
              |||:|:||:||  |||||
              ACGGGUUGUGG  AGAGU
3'-UUU...CGAUG                  CCCUC...AUG-5'
             |                  |
             38                 21        ncS63
```

```
             29                                   65    BCAL2343
             |                                    |
5'-UCG...GUCUG     GAAA    AAUGAGUAU           U  AGGAA...UGA-3'
              GAGUG     GCA         CGAAGGGGUCU GA
              |||||     |||         ||||||||||| ||
              CUCAC     CGU         GCUUCCCCAGA CU
3'-UUU...ACGAA     ACAAA                       U  AGAGC...AUG-5'
             |                                    |
             94                                   66    ncS63
```

Predicted interactions with BCAL1948

```
             39                                         80                 BCAL1948
             |                                          |
5'-UGA...GGGUC   G        A       C        C    GA   A  GCGUG...UCG-3'
              GCG ACGCGGGC GCC UCG GGCGGCGG GCUC   GG GG
              ||| |||||||| ||| ||| |||||||| ||||   || ||
              CGC UGCGCCCG CGG AGC CCGCCGCC CGAG   CC CC
3'-UCA...AAGUA   G            A                 AAA  G  AUAGC...CCC-5'
             |                                          |
             169                                        129                ncS11
```

```
             33                                              80              BCAL1948
             |                                               |
5'-UGA...UCGUG    UC     C   GGCA      C            UC       GCGUG...UCG-3'
              CGGG  GCGGA GCG    GCCUCG GGCGGCGGCG C  GAGGAGG
              ||||  ||||| :||    |||:|| :||::|::|| |  ||||||:
              GCCC  CGCCU UGC    CGGGGC UCGUUGUUGC G  CUCCUCU
   3'-UUUUUUUG           A             C          U UU       UACCA...AUG-5'
             |                                               |
             74                                              32              ncS16
```

```
             72      80                        BCAL1948
             |       |
5'-UGA...CGCUC       GCGUG...UCG-3'
              GAGGAGG
              ||||||:
              CUCCUCU
3'-UUU...AGUCC       UCGCC...AGC-5'
             |       |
             40      32                        ncS04
```

```
             72       81                       BCAL1948
             |        |
5'-UGA...CGCUC        CGUGU...UCG-3'
              GAGGAGGG
              ||||||||
              CUCCUCCC
3'-CUU...GAAUC        ACCUC...UGU-5'
             |        |
             20       11                       ncS27
```

```
            -30                              3                BCAL1948
             |                               |
5'-UGA...CCCGC G        AC       GAGAC       GGAUC...UCG-3'
              C GUGUCGGA  GGAGACG     GGGAGAU
              | :|:||:||  ||||||:     :|||||:
              G UAUAGUCU  CCUCUGU     UCCUCUG
3'-UUU...ACUUA G                 ACC         -5'
             |                               |
             28                              1                ncS03
```

```
            -19                       3               BCAL1948
             |                        |
5'-UGA...CGGAA   A  C                 GAUC...UCG-3'
              CGG GA GGAGAC    GGGAGAU
              |:| || ||||||    :|||||:
              GUC CU CCUCUG    UCCUCUG
3'-CUU...GUAUA             UACC       -5'
             |                       |
             23                      1                ncS05
```

```
            -18         -7                 BCAL1948
             |           |
5'-UGA...GGAAC           ACGGG...UCG-3'
              GGA GACGGAG
              ||| |||||||
              CCU CUGCCUC
3'-UGU...AGCGG   C       CCACG...CCG-5'
             |           |
             70          58                ncS37
```

```
             -18       -9                    BCAL1948
              |         |
5'-UGA...GGAAC         AGACG...UCG-3'
               GGAGACGG
               ||||||:|
               CCUCUGUC
3'-UUU...UACCU         -5'
              |        |
              9        1                    ncS62
```
